# Effect of Ball Weight on Speed, Accuracy, and Mechanics in Cricket Fast Bowling

**DOI:** 10.3390/sports5010018

**Published:** 2017-02-28

**Authors:** Katharine L. Wickington, Nicholas P. Linthorne

**Affiliations:** Department of Life Sciences, Brunel University London, Uxbridge, Middlesex UB8 3PH, UK; 1204487@my.brunel.ac.uk

**Keywords:** cricket, fast bowling, modified-implement training, speed training, strength training

## Abstract

The aims of this study were: (1) to quantify the acute effects of ball weight on ball release speed, accuracy, and mechanics in cricket fast bowling; and (2) to test whether a period of sustained training with underweight and overweight balls is effective in increasing a player’s ball release speed. Ten well-trained adult male cricket players performed maximum-effort deliveries using balls ranging in weight from 46% to 137% of the standard ball weight (156 g). A radar gun, bowling target, and 2D video analysis were used to obtain measures of ball speed, accuracy, and mechanics. The participants were assigned to either an intervention group, who trained with underweight and overweight balls, or to a control group, who trained with standard-weight balls. We found that ball speed decreased at a rate of about 1.1 m/s per 100 g increase in ball weight. Accuracy and bowling mechanics were not adversely affected by changes in ball weight. There was evidence that training with underweight and overweight balls might have produced a practically meaningful increase in bowling speed (>1.5 m/s) in some players without compromising accuracy or increasing their risk of injury through inducing poor bowling mechanics. In cricket fast bowling, a wide range of ball weight might be necessary to produce an effective modified-implement training program.

## 1. Introduction

In cricket fast bowling, the release speed the bowler generates has a strong influence on the outcome of the delivery. A faster release speed reduces the batsman’s decision-making time and stroke-execution time and so limits the runs scored or increases the chance of dismissing the batsman. Training with underweight and overweight implements (also known as modified-implement training) is a recognized method of increasing release speed in throwing sports [1,2,3,4,5]. Modified-implement training is sometimes used by cricket fast bowlers; however, Petersen and colleagues [5] found that a 10-week program of training with modified balls was not effective in increasing bowling speed in male club-level cricketers.

The rationale behind modified-implement training is that with underweight implements the athlete learns to perform the throwing movement faster (i.e., ‘speed training’), and with overweight implements the athlete experiences a greater load on their body which induces neuromuscular adaptations (i.e., ‘strength training’) [4]. In the study by Petersen and colleagues [5] the modified balls were up to ±25 g from the standard ball weight (156 g). Here, we suggest that this range of ball weight (±16%) is not large enough to provide a substantial training stimulus. That is, such changes in ball weight are not sufficient to substantially change the player’s bowling speed and so are not effective at increasing the player’s speed of movement or increasing the load on the player’s body.

We estimated the strength of the relationship between ball weight and bowling speed using a simple one-segment model of cricket bowling [6]. The model assumes the arm is a uniform straight rod that can rotate about the shoulder joint, and the ball is held firmly in the hand. The bowler’s muscles exert a constant torque about the shoulder joint that acts to rotate the arm. The work performed by the bowler on the arm and ball is then *Ƭ*∆*θ*, where *Ƭ* is the shoulder torque and ∆*θ* is the angular distance over which the shoulder torque acts. This work increases the rotational kinetic energy of the arm and ball. We assume the bowler releases the ball in the forward horizontal direction and the bowler is running with speed, *v*_run-up_, which adds directly to the ball release speed. If the initial angular velocity of the arm is zero, the release speed of the ball, *v*, is given by

(1) $$v\;=\;\sqrt{\frac{2T\Delta \theta }{m_{\mathrm{ball}}\;+\;\frac{m_{\mathrm{arm}}}{3}}}\;+\;v_{\mathrm{run}-\mathrm{up}},$$

where* m*_ball_ is the mass of the ball and *m*_arm_ is the mass of the bowler’s arm.

**Figure 1:**
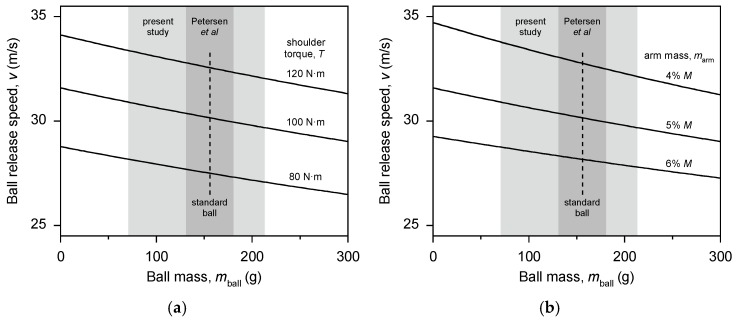
These plots show the expected decrease in ball release speed with increasing ball weight in cricket fast bowling; (**a**) effect of shoulder torque; (**b**) effect of arm mass (as % of body mass). Calculations are from a simple one-segment model of cricket bowling (equation 1) with body mass *M* = 80 kg, angular distance ∆*θ* = 270° (i.e., ¾ of a revolution), and run-up speed *v*_run-up _= 5 m/s [7]. The calculations for the effect of torque are with *m*_arm_ = 5% *M* [8], and the calculations for the effect of arm mass are with *T* = 100 N·m. The shaded areas indicate the range of ball weight used in modified-implement training studies: dark grey = Petersen et al. [5]; light grey = present study.

The bowling model indicates that ball release speed should decrease almost linearly with increasing ball weight (Figure 1). The model also indicates that the range of ball weight used in the study by Peterson and colleagues [5] would have produced only small changes in ball release speed: ±0.3 m/s, or about ±1% of the ball release speed when using a standard-weight ball. We suggest that this change in ball speed is unlikely to produce a training stimulus that is substantially different from training with a standard-weight ball. Therefore, we propose that fast bowlers should use a much greater range of ball weight when training with underweight and overweight balls.

In cricket fast bowling, accuracy can be just as important as ball speed [9]. Therefore, using a modified-implement training program with a wide range of ball weight might be counterproductive if the player’s bowling accuracy is compromised. The player’s bowling mechanics are also important in fast bowling, both for generating ball speed and for reducing the risk of injury. A fast ball release speed is associated with the angle of the front knee at front-foot contact and ball release [10,11,12,13,14], alignment of the feet relative to the direction of delivery, the angle of the shoulders at ball release, and shoulder counter-rotation during the delivery stride [11,13]. Injuries to a fast bowler’s lower back are associated with the angle of the front knee at front-foot contact and at ball release [10], and with excessive shoulder counter-rotation during the delivery stride [10,15]. Again, using a modified-implement training program with a wide range of ball weight might be counterproductive if it produces changes in the player’s bowling mechanics that reduce ball release speed or increase the player’s risk of injury.

A modified-implement training program for cricket fast bowling uses relatively inexpensive equipment and is straight-forward to apply in practice. Anecdotal evidence from cricket coaches indicates that a modified-implement training program can be effective in increasing a player’s ball release speed. Another study of the use of underweight and overweight balls in cricket fast bowling appears to be justified. The study should experimentally test the proposed theoretical relationship between ball release speed and ball weight, and then test the efficacy of a modified-implement training program, this time using a wide range of ball weight. 

The first aim of the present study was to quantify the acute effects of ball weight on ball release speed, accuracy, and bowling mechanics. This would enable the coach to make an informed decision on the most appropriate range of ball weight to use in a modified-implement training program. A group of adult male cricket players performed maximum-effort deliveries using balls with a wide range of weights, while measures of ball speed, accuracy, and mechanics were obtained using a radar gun, a bowling target, and a 2D video analysis. The second aim of the study was to investigate the effects of a period of sustained training with underweight and overweight balls on ball release speed, accuracy, and bowling mechanics. This would help the coach decide if a modified-implement training program with a wide range of ball weight is likely to be effective in increasing bowling speed without compromising accuracy or increasing the risk of injury. The cricket players were assigned to either an intervention group who trained with underweight and overweight balls, or to a control group who trained with standard-weight balls. Ball release speed, accuracy, and bowling mechanics were measured before and after the training period, and statistical techniques were used to identify changes arising from the training programs.

## 2. Methods

### 2.1. Participants

Ten adult male fast-medium pace bowlers (age, 24 ± 5 years; height, 1.80 ± 0.06 m; body mass, 90 ± 8 kg; mean ± SD) from the Counties Academy in Slough, United Kingdom, volunteered to participate in the study (Table 1). All participants were currently playing cricket at club or county level and were free from any injuries that restricted their ability to perform fast bowling. All the participants were well-conditioned and had previous experience training with underweight and overweight balls in earlier seasons. The participants had a range of bowling actions (Table 1). Participants were informed of the procedures and inherent risks prior to their involvement, and written informed consent for inclusion was obtained before participating in the study. The study was conducted in accordance with the Declaration of Helsinki and the protocol was approved by the Ethics Committee of Brunel University London (Project identification code: 0637).

### 2.2. Modified Balls

This study used standard-weight balls (156 g) and five types of modified balls (71, 113, 142, 198, and 213 g). The standard competition cricket balls (Kookaburra, Melbourne, Australia) were compliant with Marylebone Cricket Club regulations. The modified balls were custom-made and had the same dimensions and aesthetics as a standard cricket ball. The modified balls used the same four-piece outer shell with stitched seams as in a standard ball, but had filling materials of different densities so as to achieve the desired weight. The modified balls ranged in weight from 46% to 137% of the standard ball weight.

### 2.3. Test Procedures

The study was carried out over an eight-week period, which started about three months prior to the competition season. The two test sessions (‘before’ and ‘after’) took place in an indoor cricket training facility and were conducted at the same time of day, on the same day of the week, eight weeks apart. The size of the facility enabled the participants to use their full run-up lengths, which were measured and replicated in both test sessions. Before both test sessions, the participants performed a standardized warm-up that included bowling at least six deliveries at gradually increasing speed. Each participant was marked for digitizing prior to testing. Twenty-two reflective markers were secured to anatomical locations on the non-bowling side of the body (greater tuberosity, acromion process, olecranon, pisiform, fifth metacarpal head, greater trochanter, superior tibiofibular joint, lateral malleolus, styloid process, fifth metatarsal, intermetatarsal three joint, and third distal phalange).

The participants were asked to bowl as fast as possible ‘over the wicket’ and to try to hit the top of the off-stump after the bounce. In the first test session the participants bowled 13 deliveries; the first three deliveries used a standard cricket ball and the next 10 deliveries used the five modified cricket balls. The participants bowled two deliveries per modified ball and the order of ball weights was randomized. In the second test session the participants bowled 10 deliveries using a standard cricket ball. In both test sessions, the time interval between deliveries was about 60–90 s. A rest interval of this duration is sufficient to eliminate the effect of fatigue on performance in a short bowling spell [15].

Bowling speed was obtained using a Stalker Sport radar gun (Applied Concepts, Plano, TX, USA). The radar gun was placed on a tripod 3.0 m behind the middle stump and aimed at the ball release point (about 2.1 m above the wickets at the bowler’s end) [15]. The radar gun reported ball speed to the nearest mph (i.e., ±0.2 m/s), and the error in ball speed arising from misalignment of the radar gun was calculated to be less than 0.1 m/s.

**Figure 2:**
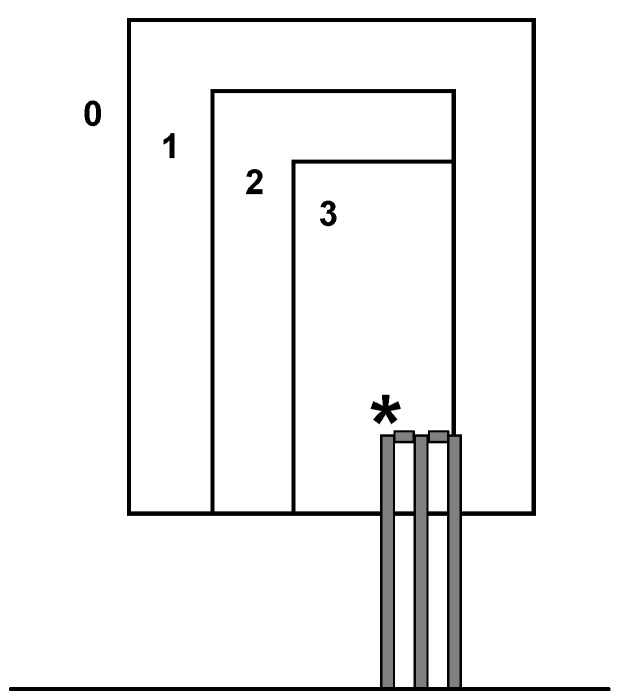
Locations of the target zones (0, 1, 2, and 3) used to score bowling accuracy (for bowling to a right-handed batsman). Participants were asked to try to hit the top of the off-stump (*) after the bounce.

Bowling accuracy was measured using a target method similar to that developed by Portus and colleagues [15]. The target consisted of three overlaying rectangles (1.00 m × 0.46 m, 1.20 m × 0.69 m, and 1.40 m × 1.15 m; height × width) placed 0.5 m beyond the stumps (Figure 2). The rectangles were scored 3, 2, and 1, with balls outside the largest rectangle receiving a score of 0. The lower edge of the target was 0.50 m above the base of the stumps and so balls delivered with too full a length received a score of 0.

Bowling mechanics were obtained from a 2D video analysis of the delivery stride [15]. A Casio EX-FH20 (Casio, Shibuya, Japan) video camera operating at 30 Hz was positioned perpendicular to the sagittal plane of the bowler at a distance of 6.0 m. This camera was used to record the participant’s knee angles at the instants of back-foot contact, front-foot contact, and ball release. A GoPro Hero4Silver H.264 (GoPro, San Mateo, CA, USA) video camera operating at 120 Hz was positioned overhead at a height of 3.7 m. This camera was used to record the angles of the shoulders and feet at the instants of back-foot contact and front-foot contact. Video images of all deliveries were analyzed using Tracker video analysis software (Open Source Physics, Davidson, NC, USA). Shoulder angle was defined by a line joining the acromion processes of the left and right shoulders [10,15], and foot angle was defined by a line joining the mid-point of the heel to the intermetatarsal three joint [15]. Knee angle was defined by lines joining the greater trochanter, superior tibiofibular joint, and lateral malleolus, and was measured relative to a straight leg (180°). Shoulder angles were measured in an anti-clockwise direction relative to the direction of bowling (the zero line) [16], and foot angles were measured in a clockwise direction.

The uncertainty due to digitizing was estimated by re-digitising a representative trial ten times. The 95% confidence interval in the foot, knee, and shoulder angles were about 1°, 1°, and 2° respectively. The greatest source of uncertainty in the knee and shoulder angles was expected to arise from the sampling frequency of the video camera, and this uncertainty was taken as one half of the difference between the value at the instant of interest and the value at one frame before the instant of interest [17]. The uncertainties were: back leg knee angle at back-foot contact, 4°; front leg knee angle at front-foot contact, 2°; front leg knee angle at ball release, 3°; shoulder angle at back-foot contact, 2°; and shoulder angle at front-foot contact, 4°.

### 2.4. Modified-Implement Training Program

The participants were randomly assigned to the two training groups. The two training groups (‘Intervention’ and ‘Control’) followed a prescribed training program of three sessions a week for eight consecutive weeks. The training programs were similar to those used in previous studies of baseball pitching and cricket fast bowling [3,5], and the bowling frequency and workload were within the guidelines of the England and Wales Cricket Board [18]. Both groups had a progressive increase in load every two weeks with an increase in the number of deliveries, and the Intervention group used a wide and contrasting range of ball weights in every session. During the training sessions for the Intervention group, the participants bowled 36–42 deliveries at maximal effort while aiming for the top of the off-stump (for a right-handed batsman) using underweight and overweight balls (Table 2). The training sessions for the Control group were identical to those for the Intervention group, except that a standard-weight ball (156 g) was always used. During the eight-week training period, no other physical or technical training was undertaken by the participants. Training was conducted in the same indoor cricket training facility as was used for the test sessions. No feedback from a coach was given to the participants during the training sessions.

### 2.5. Analysis of the Acute Effects of Ball Weight

For the first test session, data from each of the participants were analyzed separately. The ball speeds, accuracy scores, and bowling mechanics variables for the participant were plotted against ball weight, and a straight line (*y* = *ax* + *b*) and a u-shape (*y* = *y*_m_ + *c*(*x*–*x*_m_)^2^) were fitted to the data. The decision about the most appropriate curve was guided by examining the distribution of the residuals [19]. If both curves seemed appropriate for the data, a calculation of Akaike’s Information Criterion (AICc) was used to determine which of the curves gave the best fit [20]. If a straight line was the best fit to the data, the effect of ball weight on the variable was taken as the gradient of the line (*a*). If the 90% confidence interval of the gradient included zero, ball weight was deemed to have no effect on the variable [21]. If a u-shape was the best fit to the data for the variable, the maximum/minimum value of ball weight was taken as the maxima/minima in the fitted u-shape curve (*x*_m_). If the 90% CI of *x*_m_ included 156 g, the standard ball weight was deemed to be the optimum weight. In this study, a less conservative confidence interval (90%) was used so as to give a greater chance of identifying potentially beneficial or detrimental effects [22].

The bowling model was assessed using the ball speed versus ball weight data. Equation (1) was fitted to the ball speed versus ball weight data for each of the ten participants. A non-linear regression was performed using the Levenberg-Marquardt algorithm, with the angular range of the arm set to ∆*θ* = 270° and the run-up speed set to *v*_run-up_ = 5 m/s. We tested two versions of the bowling model; the first version had only one fitted variable (*T*, with *m*_arm_ set to 5% *M*), and the second version had two fitted variables (*T* and *m*_arm_). The decision about the best version of the model was guided by examining the distribution of the residuals [19] and with a calculation of Akaike’s Information Criterion [20]. The fit values for shoulder torque were expected to be similar to values measured in adult male fast bowlers [13], and the fit values for arm mass were expected to be about 5% of the participant’s body mass [8].

### 2.6. Analysis of the Modified-Implement Training Program

The efficacy of the modified-implement training program was investigated using a group analysis and an individual analysis. Group statistics are often used to compare the outcome of groups of individuals in response to an intervention program. However, an intervention program can produce considerable inter-individual differences in outcomes due to differences in biological make-up. Also, when investigating sports performance, the coach is usually more concerned with the individual case than with the group outcome [23]. Therefore, in the present study there was an individual analysis of the response of each participant to the training program, as well as analysis of the response of the group.

In this study, the smallest practically important change in ball speed was taken as 1.5 m/s, which corresponds to the smallest change that a top batsman would be expected to notice [5]. The smallest practically important change in the mean accuracy score was taken as 0.1, which corresponds to a change in accuracy score of 3 over the course of 12 deliveries (i.e., 2 overs) [5]. For bowling mechanics, the smallest practically important changes were taken as 10° for the back leg knee angle at back-foot contact, front leg knee angle at front-foot contact, and front leg knee angle at ball release, and as 20° for the back foot angle, front foot angle, shoulder angle at back-foot contact, and shoulder angle at front-foot contact. These values correspond to the smallest change that an experienced coach would be expected to notice in a player.

In this study, we estimated the typical error of measurement for each of the variables from the within-subject variations [24]. For each of the 10 participants, the standard deviation of the 10 deliveries with the standard-weight ball in the second test session was calculated, and the average of these standard deviations was taken as the typical error for the variable.

In the second test session, the participant bowled 10 deliveries with the standard-weight ball (156 g), but in the first test session, the participant bowled only three deliveries with this ball. The mean value of the three deliveries could be taken as the baseline value (i.e., ‘before’) for the variable. However, we determined the baseline value by using the curve of best fit to the variable versus ball weight data to calculate the value of the variable at a ball weight of 156 g. This method was expected to give a more accurate baseline measure because it used data from a greater number of deliveries (13 versus 3).

For the individual analysis of the participants the mean value at the two test sessions (‘before’ and ‘after’) and the difference between the two test sessions (‘difference’) was calculated for each variable. The magnitude of this difference was interpreted as ‘beneficial’, ‘trivial’, or ‘detrimental’ using the likely limits method for assessing a change in a performance test by an individual [22,25]. A less conservative confidence interval of 90% was used to give a greater chance of identifying potentially beneficial or detrimental effects [22]. Because the difference between the two test sessions involved two measurements, the typical error in the difference was taken as √2 times the typical error in the variable [26].

For the group analysis a repeated-measures *t*-test was conducted on the two test sessions (‘before’ and ‘after’) using IBM SPSS Statistics version 20 (IBM, Armonk, NY, USA). The significance level was set to a less conservative value of *α* = 0.10 to give a greater chance of identifying potentially beneficial or detrimental effects [22]. For each variable the mean difference, 90% CI, *p*-value, within-pair correlation, and effect size (Cohen’s *d*) were calculated. The mean differences in the *t*-tests were interpreted using the 90% confidence intervals and *p*-values [21]. In this study the primary outcome variable was ball speed. Accuracy and the bowling technique variables were secondary outcome variables and so it was not essential to apply a correction for multiple comparisons [21]. A *t*-test can be used with extremely small sample sizes (≤5), and acceptable statistical power (>80%) can be achieved as long as the effect size is large and the within-pair correlation is high [27]. In the present study the effect size for a practically important change in ball speed (1.5 m/s) was expected to be about 1.5 (assuming a standard deviation in the change in ball speed of about 1.0 m/s). The statistical power of the present study to detect a practically important change in ball speed was therefore expected to be about 0.95 (G*Power version 3.1; Heinrich-Heine-Universität Düsseldorf, Düsseldorf, Germany).

## 3. Results

### 3.1. Acute Effects of Ball Weight

**Figure 3:**
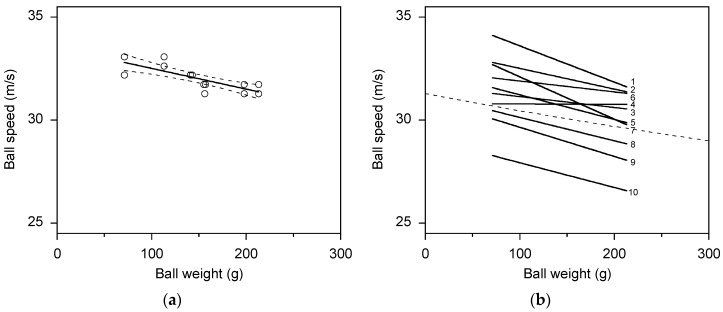
Plot (**a**) shows the linear decrease in ball speed with increasing ball weight. Data for Participant 2. The solid line is a linear regression fit and the dashed lines show the 90% confidence bands. Plot (**b**) shows the differences in the rate of decrease in ball speed among the 10 participants. Only the regression lines are shown; data points have been omitted for clarity. The dashed line shows the relationship calculated from the bowling model (Equation (1), with *T* = 110 N·m, ∆*θ* = 270°, *v*_run-up _= 5 m/s, *m*_arm_ = 5% *M*, and *M* = 90 kg).

The ball speed generated by a participant tended to decrease as ball weight increased (Figure 3 and Supplementary Data 1). For seven of the ten participants, a straight line was the best fit to the data; for two participants, a u-shape was equally as good as a straight line; and for one participant, a u-shape was a better fit than a straight line. A straight line was therefore taken as the most appropriate relationship between ball speed and ball weight. The rate of decrease in ball speed was about 1.1 m/s per 100 g increase in ball weight (Table 3), but this rate varied among the participants, and three participants (3, 4, and 6) showed no clear effect of ball weight on ball speed.

The bowling model (Equation (1)) also gave a good fit to the ball speed data (Table 3 and Supplementary Data 2). The AICc values indicated that the version of the model with one fitted variable (*T*) was the most appropriate for seven participants, and the version with two fitted variables (*T* and *m*_arm_) was the most appropriate for two participants. However, for the version with one fitted variable, the distribution of residuals was not uniform for three participants. Therefore, the version with two fitted variables (*T* and *m*_arm_) was deemed to be a slightly more appropriate model. For both versions of the model the values for the coefficient of variation, root-mean-square deviation, and AICc were similar to those for the linear fit. The three participants (3, 4, and 6) that showed no clear effect of ball weight on ball speed had large uncertainties in the fitted values for shoulder torque and arm mass (Table 3).

Bowling accuracy tended to be independent of the weight of the ball (see Supplementary Data 1). For six of the ten participants a straight line was the best fit to the data and for four participants a u-shape was equally as good as a straight line. A straight line was therefore taken as the most appropriate relationship between bowling accuracy and ball weight. For eight of the ten participants the gradient of the line was not substantially different from zero. For participants 6 and 8, bowling accuracy tended to be reduced when using the two overweight balls. Overall, we concluded that accuracy was not adversely affected when bowling with underweight and overweight balls. Across all ball weights the mean accuracy scores for the participants ranged from 1.5 to 2.2.

Bowling mechanics also showed a strong tendency to be independent of the weight of the ball (see Supplementary Data 1). For shoulder angle at back-foot contact a straight line was the best fit to the data for a clear majority of participants and the gradient of the line was not substantially different from zero. Similar null results were obtained for the shoulder angle at front-foot contact, back foot angle, front foot angle, back leg knee angle at back-foot contact, front leg knee angle at front-foot contact, and front leg knee angle at ball release.

### 3.2. Effects of the Modified-Implement Training Program

The typical error for the variables (calculated from the 10 deliveries with the standard-weight ball in the second test session) was as follows: ball speed, 0.6 m/s; accuracy score, 0.6; back foot angle, 7°; front foot angle, 10°; back leg knee angle at back-foot contact, 4°; front leg knee angle at front-foot contact, 5°; front leg knee angle at ball release, 6°; shoulder angle at back-foot contact, 8°; and shoulder angle at front-foot contact, 7° (see Supplementary Data 3). For the baseline values (i.e., the first test session) the uncertainty in the variable value calculated from the mean of the three deliveries with the standard-weight ball was slightly greater than that calculated from the curve of best fit to the variable versus ball weight data. This confirmed the decision to use the variable values calculated from the second method as the baseline values. All participants in the Intervention group completed both test sessions (*n* = 5), but two participants in the Control group did not attend the second test session (*n* = 3).

The individual analysis of the participants showed that the Intervention training program was partially successful in increasing ball speed (Table 4 and Supplementary Data 4). Two of the five participants in the Intervention group (participants 1 and 3) showed a beneficial increase in ball speed, but three participants showed only trivial changes. All three participants in the Control group showed only trivial changes in ball speed.

For bowling accuracy, six participants showed what initially appeared to be a substantial increase in score (0.3–1.2) after completing the training program (see Supplementary Data 4). However, the typical error in the accuracy score (0.6) was much greater than the smallest worthwhile change (0.1) and so the target method [5] used in the present study was not able to reliably identify small changes in bowling accuracy. The accuracy results were interpreted as almost certainly beneficial for participant 1, probably beneficial for participant 3, and unclear for the other participants.

The Intervention and Control training programs did not induce detrimental changes in the participant’s bowling mechanics (see Supplementary Data 4). The values of the bowling mechanics variables were similar to those reported in previous studies (after taking into account the participant’s bowling action) [10,13,15,28]. The range of mean values from the participants were as follows: back foot angle, 37° to 109°; front foot angle, –7° to 34°; back leg knee angle at back-foot contact, 141° to 176°; front leg knee angle at front-foot contact, 148° to 179°; front leg knee angle at ball release, 125° to 178°; shoulder angle at back-foot contact, 11° to 79°; and shoulder angle at front-foot contact, 18° to 77°. After completing the training program the participants in both the Intervention and Control groups showed only trivial changes in front foot angle, back leg knee angle at back-foot contact, front leg knee angle at front-foot contact, front leg knee angle at ball release, and shoulder angle at back-foot contact. A few participants showed a probable or almost certain change in back foot angle, front foot angle, shoulder angle at back-foot contact, or shoulder angle at front-foot contact. However, these changes were not clearly associated with one or the other of the training groups.

The results from the group analysis were consistent with those obtained in the individual analysis (see Supplementary Data 4). There was a substantial and statistically significant increase in ball speed in the Intervention group (1.0 m/s), but the change was interpreted as possibly trivial and unlikely to be detrimental (14/86/0; beneficial/trivial/detrimental) (Before: mean = 31.4 m/s, SD = 0.9 m/s; After: mean = 32.3 m/s, SD = 1.4 m/s; Difference: mean = 1.0 m/s, SD = 0.9 m/s, *t*(4) = 2.4, *p* = 0.07, inter-pair correlation = 0.77, Cohen’s *d* = 1.1). The small change in ball speed in the Control group (0.2 m/s) was not statistically significant and was interpreted as possibly trivial (6/90/4). Bowling accuracy in the Intervention group showed a substantial beneficial increase, but the change in the Control group was unclear. None of the bowling technique variables showed statistically significant changes and the magnitudes of the changes were interpreted as trivial or unclear.

## 4. Discussion

This study found that ball speed tended to decrease as ball weight increased, at a rate of about 1.1 m/s per 100 g increase in ball weight. Bowling accuracy and bowling mechanics were not adversely affected when using underweight and overweight balls. Two of the five participants in the Intervention group showed a beneficial increase in ball speed and so the modified-implement training program used here might be an effective method of training for some adult male fast bowlers. The modified-implement training program did not compromise the bowling accuracy of the participants in the Intervention group, nor was there substantial evidence that the training program increased the risk of injury to the participants through inducing detrimental changes in bowling mechanics.

### 4.1. The Acute Effects of Ball Weight

The values of ball release speed in the present study (with the standard-weight ball) were similar to those reported in previous studies of cricket fast bowling [10,12,15,28]. Likewise, the values of the bowling mechanics variables in the present study (after taking into account the participant’s bowling action) were similar to those reported in previous studies [10,15,28]. These similarities suggest that the findings from the present study regarding the effects of ball weight are likely to apply to most other adult male fast bowlers.

The shoulder torque values obtained from the bowling model fits are similar to those measured in adult male fast bowlers (50–100 N·m) [13], and the arm mass values are in modest agreement with those expected from the body mass of the participants (3.9–5.1 kg, assuming arm mass is 5% of body mass [8]). This suggests that the bowling model contains the essential features of cricket fast bowling. However, the bowling model does not include shoulder counter-rotation and trunk flexion during the delivery stride, the action of the non-bowling arm, and flexion of the hand and fingers. These factors can make substantial contributions to ball speed [28,29], and so the bowling model might overestimate the shoulder torque required to achieve a given ball release speed by about a factor of two.

Previous studies of fast bowlers reported run-up speeds of 4–6 m/s [7]. Although run-up speed is positively associated with ball release speed [28,30], experienced fast bowlers maintain a highly consistent run-up speed when bowling a series of overs [10]. In the present study, the participant’s run-up speed was not measured. However, the participant’s run-up length was held constant, both during a test session and between test sessions. Therefore, the participant very likely maintained a constant run-up speed and so the observed changes in ball speed, accuracy, and bowling mechanics are not likely to be due to systematic changes in run-up speed.

**Figure 4:**
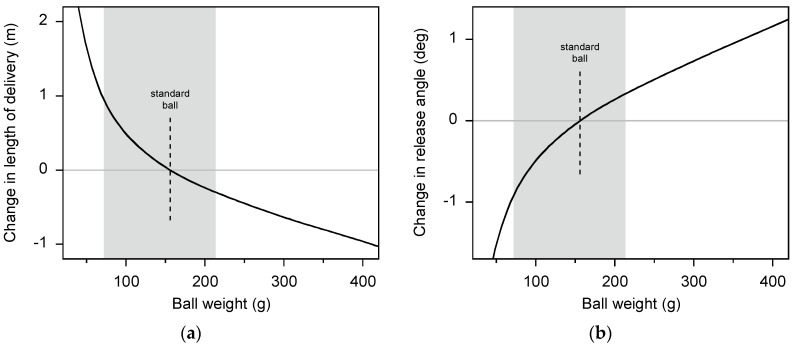
These plots show the effect of ball weight on the ability of a player to bowl a ‘good length’; (**a**) change in the length of the delivery, (**b**) change in release angle required to maintain the same length as that achieved with a standard-weight ball (156 g). Calculations are from a 2D aerodynamic model of the flight of a cricket ball [31]. The shaded area indicates the range of ball weight used in the present study.

In the present study we investigated whether bowling with underweight and overweight balls has an adverse effect on bowling accuracy. A crucial aspect of accurate fast bowling is to bowl a ‘good length’, where the ball hits the ground about 6–7 m in front of the stumps. To address this issue further, we used a 2D aerodynamic model of the flight of a cricket ball [31] to calculate the effect of changes in ball weight on the length of the delivery. The calculations were for a ball release speed of 30 m/s (with the standard-weight ball), a release angle of –6.5°, a drag coefficient of 0.5, and a lift coefficient of 0.15. The rate of decrease in ball speed with ball weight was taken as 1.1 m/s per 100 g. Our calculations showed that changes in the length of the delivery are mainly due to the change in ball release speed (arising from the change in ball weight) and the effect of aerodynamic lift (arising from ball spin). For balls weighing more than about 80 g the changes in length are relatively minor (Figure 4a). However, below about 80 g aerodynamic lift becomes increasingly important and produces a substantial increase in the length of the delivery (>0.8 m). Therefore, we warn coaches that a modified-implement training program that uses balls weighing less than about 80 g might produce an excessive change in the player’s bowling action (especially ball release angle) if the player attempts to bowl a good length with this ball (Figure 4b).

### 4.2. The Effects of a Modified-Implement Training Program

Our modified-implement training program used ball weights that ranged from 46% to 137% of the standard ball weight. However, the results from the eight-week modified-implement training program provided only partial support for the efficacy of the training program. In the individual analysis the modified-implement training program induced a beneficial increase in ball speed in only two of the five participants. A modified-implement training program for cricket fast bowling uses relatively inexpensive equipment and is straight-forward to apply in practice. Also, there was no evidence from the present study that a modified-implement training program produces a reduction in ball release speed or detrimental changes in bowling technique. Therefore, we recommend that fast bowlers use a modified-implement training program because there does not appear to be much to lose and there is potential for a substantial gain in performance.

In the present study we took care to control potential confounding factors between the two test sessions (such as the venue, equipment, testing protocol, personnel, time of day, and air temperature). However, other factors (such as the motivation of the participants) might have contributed to the observed changes in ball release speed, accuracy, and bowling technique variables. Using a greater number of participants would have increased the confidence in our results from both the individual and group analyses. Also, we might have observed a practically meaningful increase in ball speed in more than two of the participants if the training program was conducted over a greater length of time (e.g., 16 weeks rather than 8 weeks). 

In the present study, we might have observed larger increases in ball speed in the participants if the training program was conducted with a greater range of ball weight. We suspect that changes in release speed of at least ±5% might be necessary for a modified-implement training program to be effective. This proposal is supported by results from studies of other throwing sports. Training with underweight and overweight implements is a recognized method of increasing throwing speed in baseball, handball, water polo, javelin throw, discus throw, and shot put [1,2,3,4]. Coaches in these sports recommend training with implements that differ by 5%–20% from the standard implement weight. This range of implement weight produces substantial changes in release speed and, presumably, provides a sufficient training stimulus to the athlete. For instance, changing the weight of a handball by ±20% changes the ball release speed by about ±4% [32,33] and changing the weight of the shot by ±20% changes the release speed by about ±9% [34].

The weight of a baseball (142 g) is similar to that of a cricket ball (156 g), but the effect of ball weight on ball release speed is about three times greater in baseball pitching than in cricket fast bowling. This difference probably arises because baseball pitching and cricket fast bowling use different throwing actions (arm bending at the elbow versus straight-arm action). In cricket fast bowling, the ball release speed decreases at a rate of about 1.1 m/s per 100 g increase in ball weight, whereas in baseball pitching, the ball release speed decreases according to *v* = *A*/*m*_ball_^0.15^, where *A* is a constant [6,35], and so ball release speed decreases at a rate of about 3.6 m/s per 100 g increase in ball weight. Therefore, a modified-implement training program for cricket fast bowling should require a much greater range of ball weight than that for a baseball pitching training program.

In the present study, the maximum changes in ball weight of –54% and +37% produced changes in ball speed of about +0.9 m/s and –0.6 m/s (assuming a rate of decrease in ball speed of 1.1 m/s per 100 g). However, these changes in ball speed are only about +3% and –2% of the speed achieved with the standard-weight ball and so might not provide a sufficiently strong training stimulus to the player. We suggest that in order to provide an effective training stimulus to the player, cricket coaches should use a range of ball weight even greater than that used in the present study. However, we do not recommend training with balls lighter than about 80 g because aerodynamic effects increase the length of the delivery too much compared to that for a standard-weight ball. Training with balls heavier than 213 g might be appropriate, but for balls heavier than about 400 g the length of the delivery is reduced by over 0.9 m, which, again, might be too different from that for a standard-weight ball. If the coach decides to use balls heavier than those used in the present study, we recommend checking for detrimental changes in the player’s bowling mechanics, particularly in the shoulders and trunk.

## 5. Conclusions 

The players in this study were able to bowl successfully at maximum effort while using a wide range of ball weight (46% to 137% of the standard ball weight). Ball speed tended to decrease as ball weight increased, at a rate of about 1.1 m/s per 100 g increase in ball weight. Accuracy and bowling mechanics were not adversely affected when using the underweight and overweight balls. The 8-week training program with underweight and overweight balls might have produced a practically meaningful increase in bowling speed (>1.5 m/s) in some players without compromising accuracy or increasing the player’s risk of injury. However, in order to apply a more effective training stimulus to the player, we recommend using balls considerably heavier (up to 400 g) than the heaviest ball used in the present study (213 g).

The strength of the dependence of ball speed on ball weight can vary substantially among the various throwing sports. When designing a modified-implement training program for a specific throwing sport, the coach should base the choice of the range of implement weight on the magnitudes of the changes in release speed that are induced by the modified implements, rather than employing a ±20% change in the implement weight, which has been recommended for some sports. A change in release speed of at least ±5% might be required in an effective modified-implement training program.

## Figures and Tables

**Table 1:** Characteristics of the participants.

Training Group	Participant	Age (Years)	Height (m)	Mass (kg)	Bowling Action^ 1^
Intervention	1	27	1.77	81	side-on
(under and overweight balls)	2	26	1.84	93	mixed
	3	23	1.87	94	front-on
	4	26	1.80	102	semi-open
	5	18	1.82	99	mixed
Control	6	19	1.91	87	mixed
(standard-weight balls)	7	20	1.80	84	semi-open
	8	20	1.73	88	mixed
	9	25	1.79	78	front-on
	10	34	1.71	97	front-on

**^1^** Identification of the participant’s bowling action was based on a visual inspection of the orientation of the back foot, hips, and shoulders during the delivery stride [16].

**Table 2:** Training program for the Intervention group (underweight and overweight balls).

Training Weeks	Session	Total Number of Deliveries	Ball Weight^ 1^	Number of Deliveries for Each Ball Weight
1–2	1	36	C–A–B–A; F–A	4–8–4–8; 4–8
	2	36	C–E–C–F; E	5–10–5–10; 6
	3	36	C–A–B–A; F–A	4–8–4–8; 4–8
3–4	1	38	C–A–B–A; A	5–10–5–10; 8
	2	38	C–E–C–F; E	4–8–4–8; 14
	3	38	C–A–B–A; A	5–10–5–10; 8
5–6	1	40	B–E–B–F; F	4–8–4–8; 16
	2	40	C–A–C–A; F	6–12–6–12; 4
	3	40	B–E–B–F; F	4–8–4–8; 16
7–8	1	42	B–E–B–F; F	4–8–4–8; 18
	2	42	C–A–C–A; F–A	6–3–6–3; 16–8
	3	42	B–E–B–F; F	4–8–4–8; 18

**^1^** Key to ball weights: A = 71 g, B = 113 g, C = 141 g, D = 156 g (not used), E = 198 g, F = 213 g. A semi colon (;) indicates a 10-minute recovery where the participant performed controlled dynamic stretches before continuing the training program.

**Table 3:** The acute effects of ball weight on ball speed (±90% CI).

	Linear Fit ^1^			Cricket Bowling Model ^2^	
Participant	Rate of Decrease in Ball Speed (m/s per 100 g)	*r* ^2^	RMSD (m/s)	Shoulder Torque *T* (N·m)	Arm Mass *m*_arm_ (kg)
1	1.8 ± 1.8	0.23	5.4	60 ± 60	1.9 ± 2.1
2	1.0 ± 0.4	0.61	1.3	100 ± 40	3.6 ± 1.7
3	0.5 ± 0.6	0.21	1.7	180 ± 190	7.0 ± 7.9
4	0.0 ± 1.0	<0.01	3.0	—	—
5	1.2 ± 0.3	0.86	0.8	80 ± 20	2.8 ± 0.7
6	0.5 ± 1.0	0.08	3.0	190 ± 360	7.3 ± 14.2
7	2.1 ± 0.6	0.75	2.0	40 ± 10	1.4 ± 0.5
8	1.1 ± 0.6	0.48	2.0	70 ± 40	3.0 ± 2.0
9	1.4 ± 0.9	0.44	2.7	50 ± 30	2.1 ± 1.5
10	1.2 ± 0.7	0.48	2.1	50 ± 30	2.3 ± 1.5

**^1^** *r*^2^ = coefficient of variation; RMSD = root-mean-square deviation. ^2 ^Equation (1), with the angular range of the arm set to ∆*θ* = 270° and the run-up speed set to *v*_run-up _= 5 m/s.

**Table 4:** Individual analysis of the effect of the training program on the participant’s ball speed.

Training Group	Participant	Before (m/s)	After (m/s)	Difference(m/s)	±90% CI (m/s)	Interpretation
Intervention	1	32.6	34.7	2.1	0.9	Probably beneficial
	2	32.0	31.7	–0.3	0.3	Probably trivial
	3	30.9	32.5	1.7	0.5	Probably beneficial
	4	30.8	31.6	0.8	0.6	Possibly trivial
	5	30.6	31.2	0.6	0.2	Probably trivial
Control	6	31.6	—	—	—	—
	7	31.0	30.2	–0.8	0.4	Possibly trivial
	8	29.5	—	—	—	—
	9	28.9	29.6	0.8	0.8	Possibly trivial
	10	27.3	27.9	0.6	0.6	Probably trivial

## Supplementary Materials

The following are available online at http://www.mdpi.com/2075-4663/5/1/18/s1; Supplementary Data 1 (Ball Weight), Supplementary Data 2 (Bowling Model Fits), Supplementary Data 3 (After), Supplementary Data 4 (Effect of Training).
