# Supplementary material for: Effect of Ball Weight on Speed, Accuracy, and Mechanics in Cricket Fast Bowling
Source: Sports (Basel). 2017 Feb 28;5(1):18. doi: 10.3390/sports5010018 (PMC5969017; doi:10.3390/sports5010018)
Supplement: Supplementary file 1 [file sports-05-00018-s001.zip › Supplementary Data 2 (Bowling Model Fits).pdf]

BOWLING MODEL **with fitted Shoulder Torque (m_{arm} = 5% M; $\Delta\theta$ = 270°; $v_{run\text{-}up}$ = 5 m/s)**

Participant 1

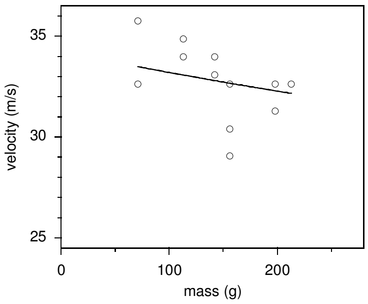

Participant 2

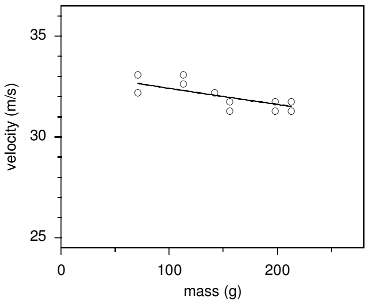

Participant 3

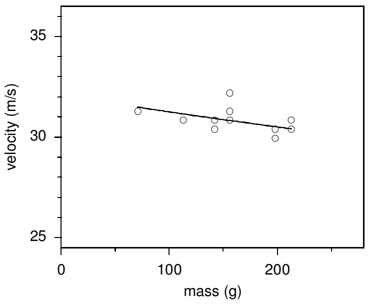

Participant 4

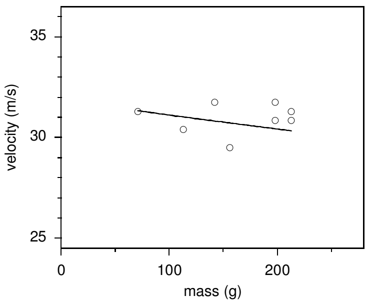

Participant 5

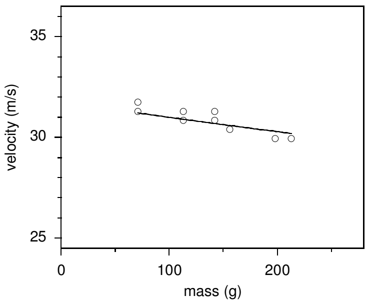

Participant 6

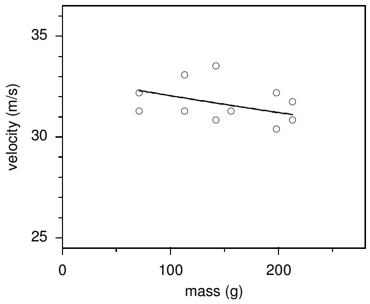

Participant 7

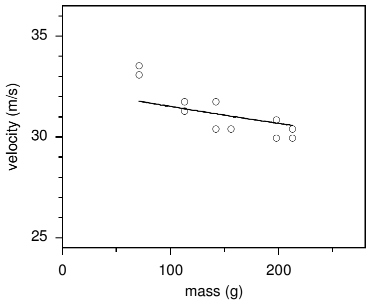

Participant 8

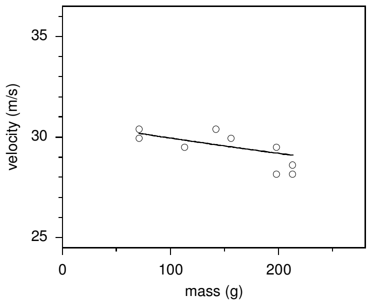

Participant 9

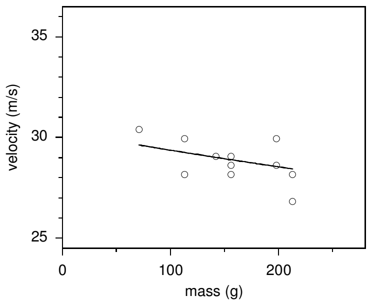

Participant 10

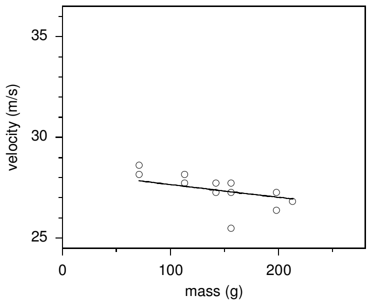

BOWLING MODEL **with fitted Shoulder Torque and Arm Mass ($\Delta\theta$ = 270°; $v_{run\text{-}up}$ = 5 m/s)**

Participant 1

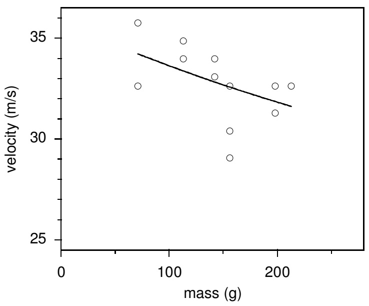

Participant 2

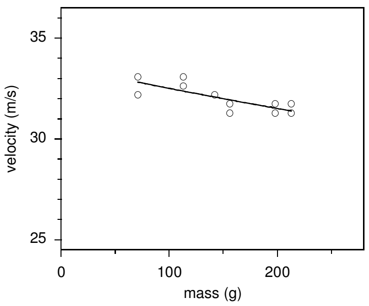

Participant 3

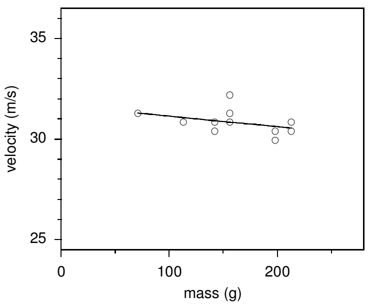

Participant 4

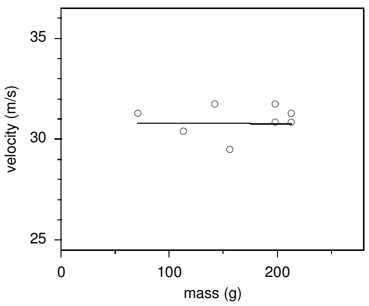

Participant 5

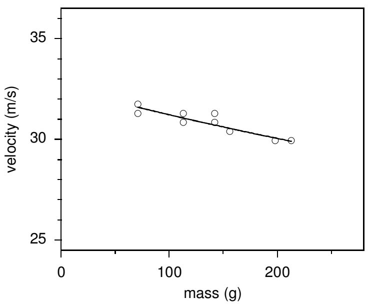

Participant 6

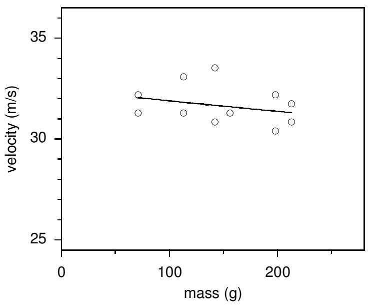

Participant 7

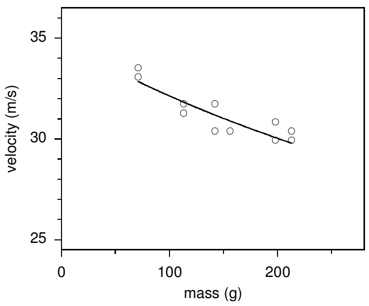

Participant 8

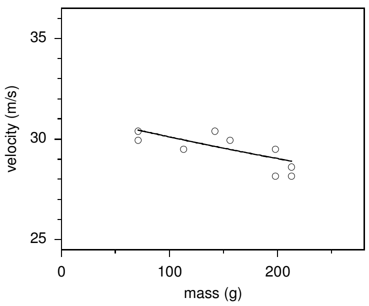

Participant 9

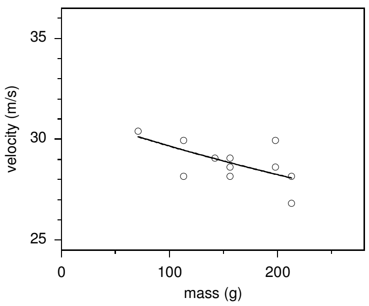

Participant 10

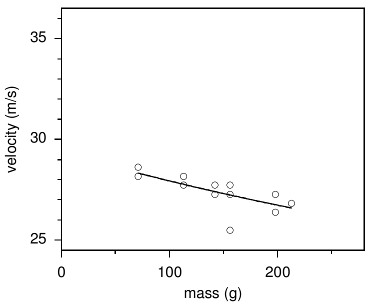

BOWLING MODEL FITS TO BALL SPEED vs BALL WEIGHT DATA

Bowling Model with one fitted variable: Shoulder Torque (T)
(m_{arm} = 5% M; $\Delta\theta$ = 270°; $v_{run\text{-}up}$ = 5 m/s)

Participant	Cricket Bowling Model 1			
	Shoulder torque T (N·m)	r^2	RMSD (m/s)	AICc
1	122 ± 7	0.18	5.6	16.57
2	132 ± 2	0.59	1.4	–19.77
3	122 ± 2	0.16	1.8	–13.19
4	130 ± 5	0.14	3.2	1.80
5	125 ± 2	0.72	1.1	–24.81
6	120 ± 4	0.05	3.0	0.67
7	112 ± 3	0.49	2.8	–1.10
8	103 ± 3	0.42	2.1	–8.86
9	88 ± 3	0.36	2.9	–0.81
10	93 ± 3	0.38	2.3	–6.81

Bowling Model with two fitted variables: Shoulder Torque (T) and Arm Mass (m_{arm})
($\Delta\theta$ = 270°; $v_{run\text{-}up}$ = 5 m/s)

Participant	Cricket Bowling Model 2				
	Shoulder torque T (N·m)	Arm mass m_{arm} (kg)	r^2	RMSD (m/s)	AICc
1	60 ± 60	1.9 ± 2.1	0.24	5.4	19.09
2	100 ± 40	3.6 ± 1.7	0.59	1.3	–17.13
3	180 ± 190	7.0 ± 7.9	0.21	1.7	–10.39
4	5000 ± 150000	210 ± 6200	<0.01	3.0	3.53
5	80 ± 20	2.8 ± 0.7	0.86	0.8	–30.01
6	190 ± 360	7.3 ± 14.2	0.08	3.0	3.72
7	40 ± 10	1.4 ± 0.5	0.78	1.9	–8.42
8	70 ± 40	3.0 ± 2.0	0.46	2.0	–6.32
9	50 ± 30	2.1 ± 1.5	0.44	2.7	0.94
10	50 ± 30	2.3 ± 1.5	0.49	2.1	–5.97

BOWLING MODEL FITS TO BALL SPEED vs BALL WEIGHT DATA

Relative probability of model

Participant	Model with one fitted variable: T (%)	Model with two fitted variables: T and m_{arm} (%)
1	78	22
2	79	21
3	80	20
4	70	30
5	7	93
6	82	18
7	3	97
8	78	22
9	71	29
10	60	40

Linear Fit

Participant	Linear Fit			
	Rate of decrease in ball speed (m/s per 100 g)	r^2	RMSD (m/s)	AICc
1	1.8 ± 1.8	0.23	5.4	19.26
2	1.0 ± 0.4	0.61	1.3	–17.14
3	0.5 ± 0.6	0.21	1.7	–10.41
4	0.0 ± 1.0	<0.01	3.0	3.53
5	1.2 ± 0.3	0.86	0.8	–30.33
6	0.5 ± 1.0	0.08	3.0	3.71
7	2.1 ± 0.6	0.75	2.0	–6.58
8	1.1 ± 0.6	0.48	2.0	–6.78
9	1.4 ± 0.9	0.44	2.7	1.08
10	1.2 ± 0.7	0.48	2.1	–5.75
