# Supplementary material for: Effect of Ball Weight on Speed, Accuracy, and Mechanics in Cricket Fast Bowling
Source: Sports (Basel). 2017 Feb 28;5(1):18. doi: 10.3390/sports5010018 (PMC5969017; doi:10.3390/sports5010018)

BALL SPEED (AFTER)

Participant 1

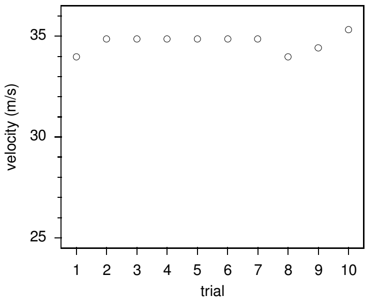

Participant 2

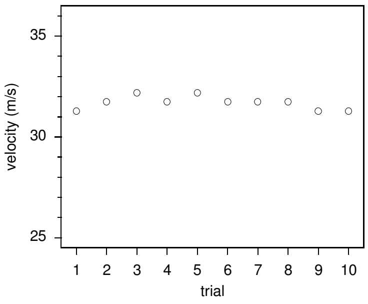

Participant 3

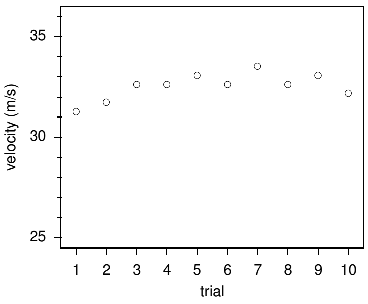

Participant 4

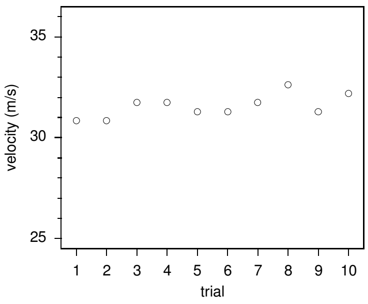

Participant 5

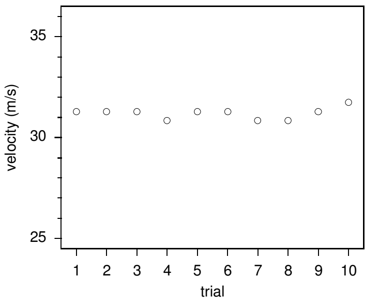

Participant 6

Participant 7

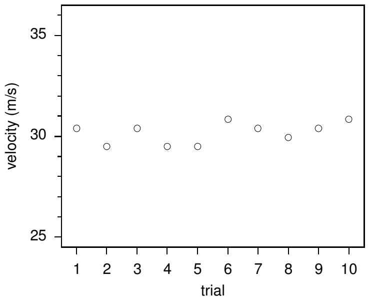

Participant 8

Participant 9

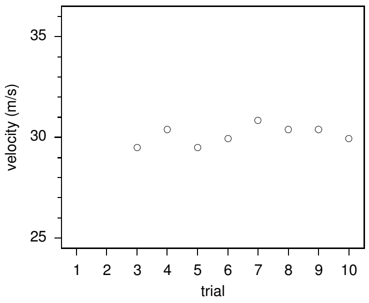

Participant 10

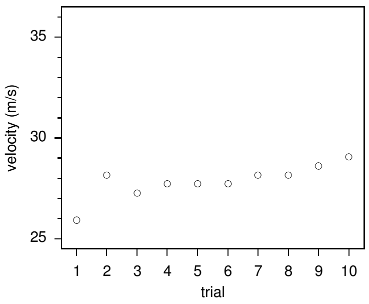

ACCURACY (AFTER)

Participant 1

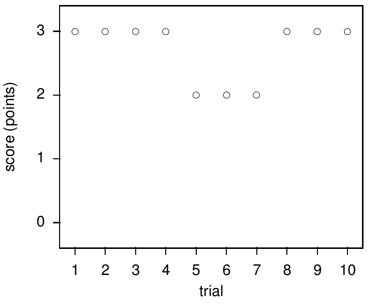

Participant 6

Participant 2

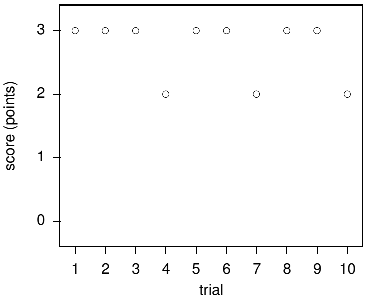

Participant 7

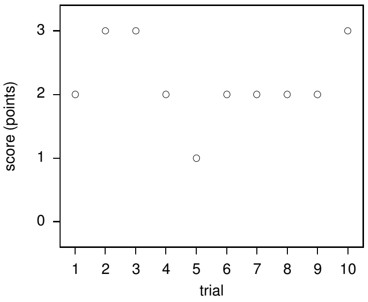

Participant 3

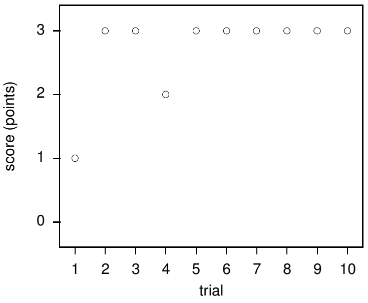

Participant 8

Participant 4

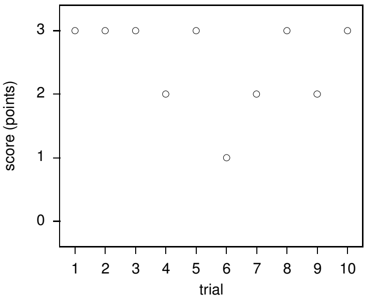

Participant 9

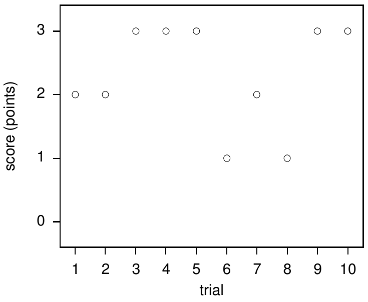

Participant 5

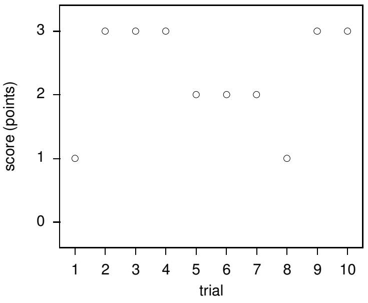

Participant 10

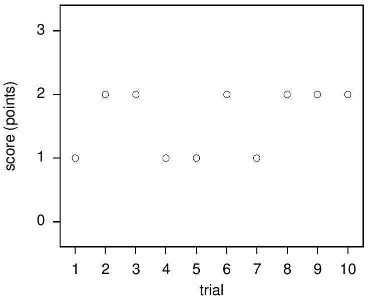

BACK FOOT ANGLE (AFTER) *

Participant 1

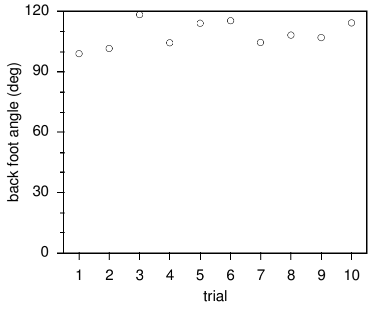

Participant 2

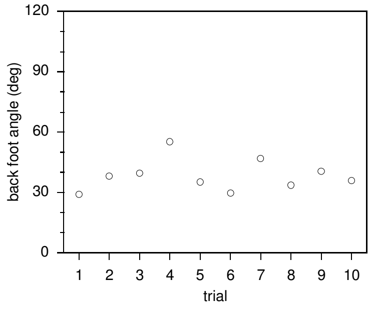

Participant 3

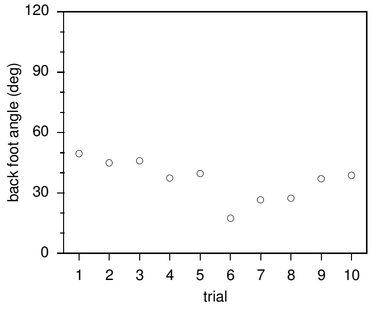

Participant 4

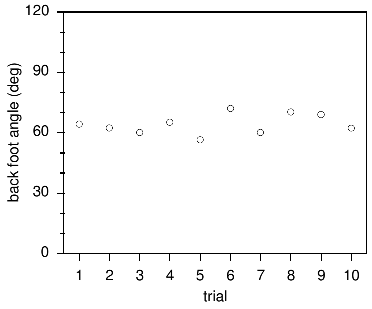

Participant 5

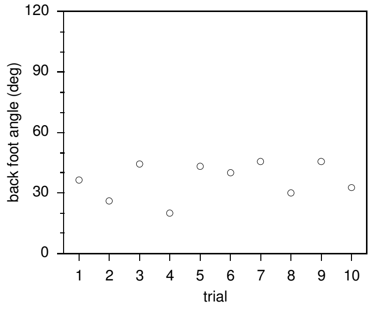

Participant 6

Participant 7

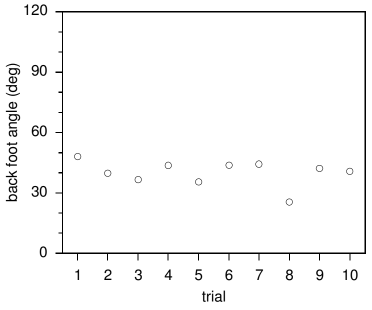

Participant 8

Participant 9

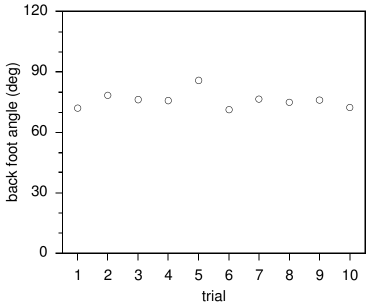

Participant 10

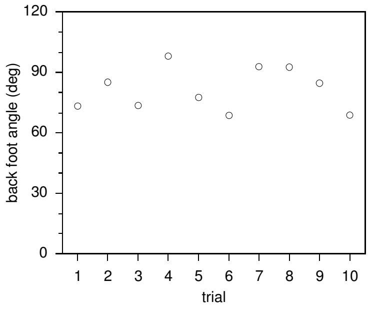

* Foot angle measured in clockwise direction relative to direction of run-up

FRONT FOOT ANGLE (AFTER) *

Participant 1

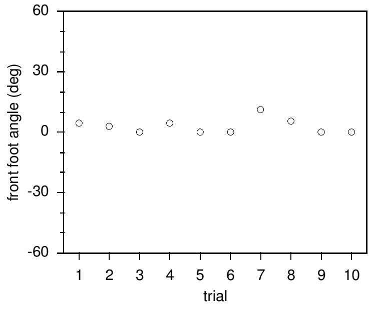

Participant 2

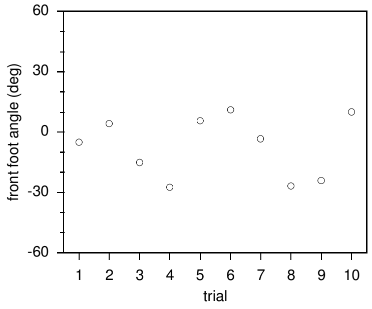

Participant 3

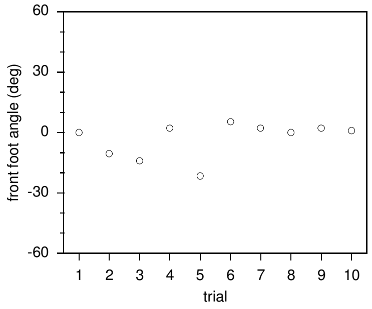

Participant 4

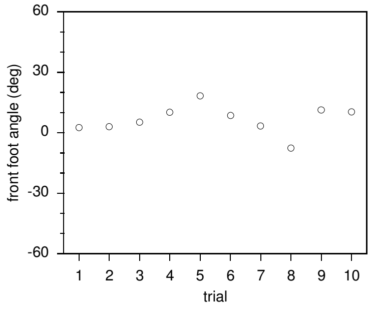

Participant 5

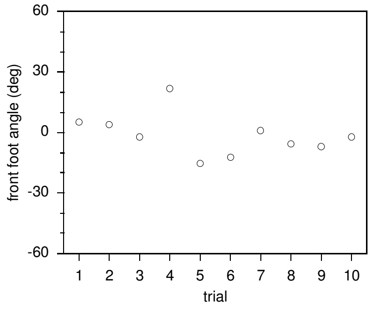

Participant 6

Participant 7

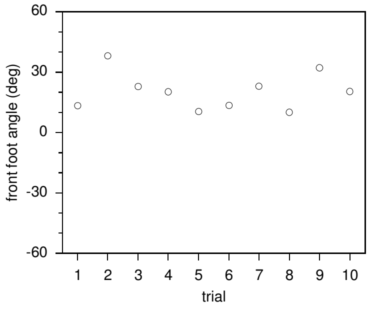

Participant 8

Participant 9

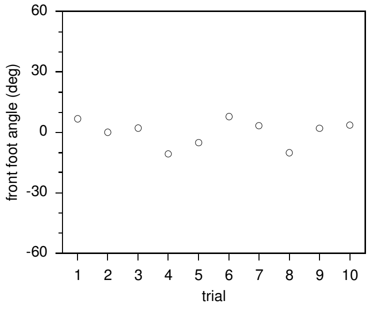

Participant 10

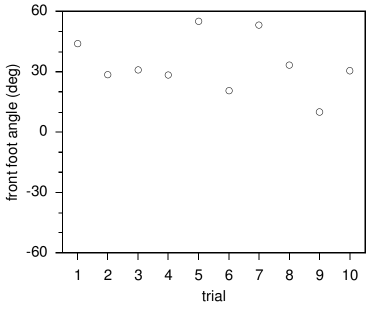

* Foot angle measured in clockwise direction relative to direction of run-up

KNEE ANGLE (BACK FOOT CONTACT) (AFTER)

Participant 1

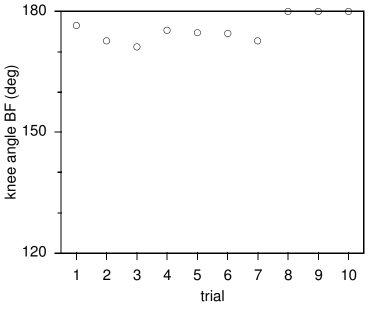

Participant 2

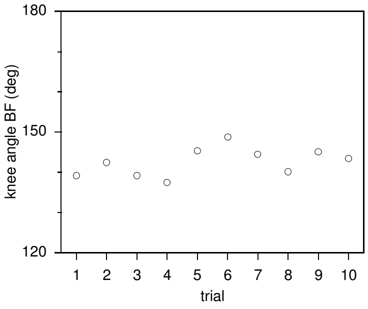

Participant 3

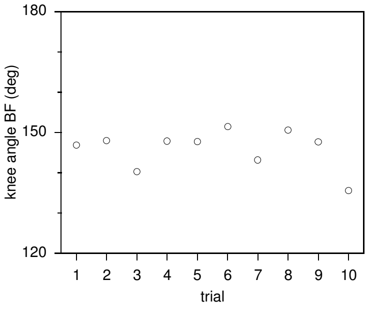

Participant 4

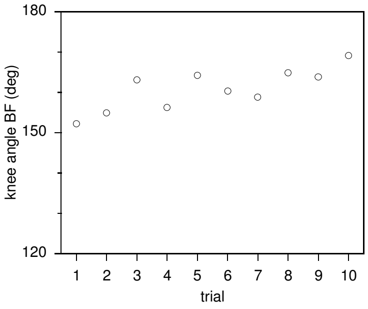

Participant 5

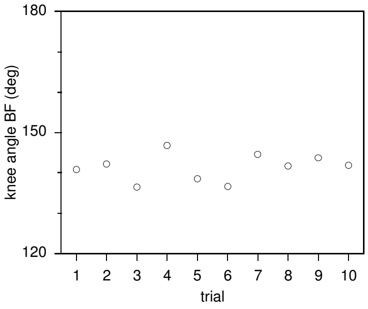

Participant 6

Participant 7

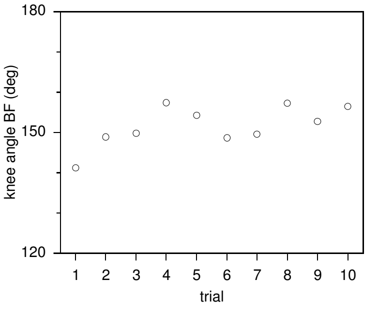

Participant 8

Participant 9

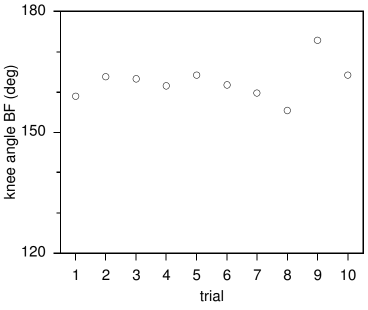

Participant 10

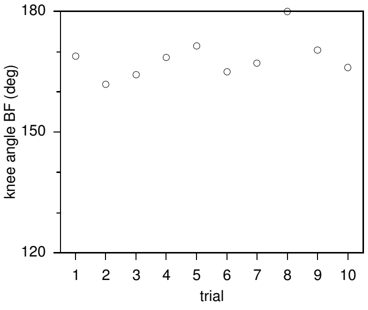

KNEE ANGLE (FRONT FOOT CONTACT) (AFTER)

Participant 1

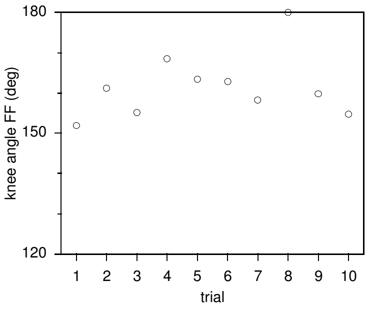

Participant 2

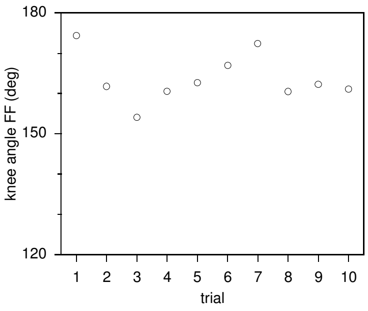

Participant 3

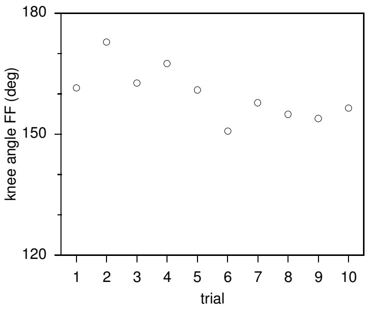

Participant 4

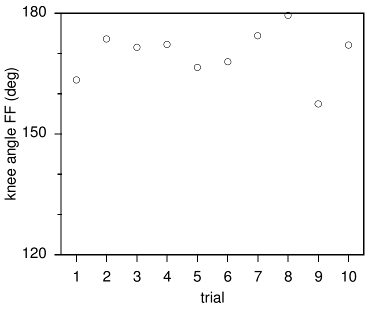

Participant 5

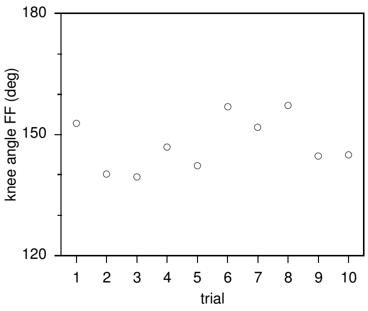

Participant 6

Participant 7

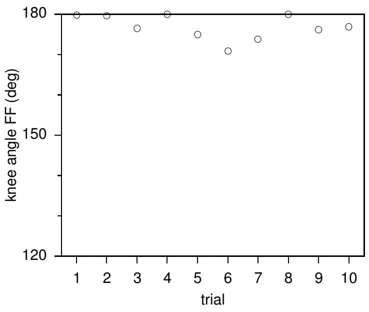

Participant 8

Participant 9

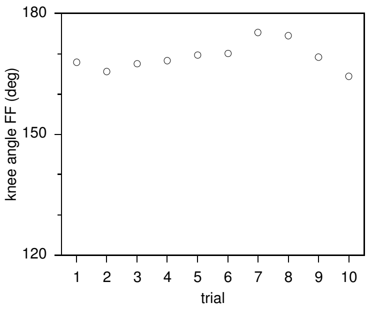

Participant 10

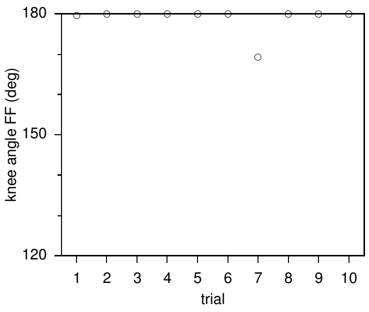

KNEE ANGLE (RELEASE) (AFTER)

Participant 1

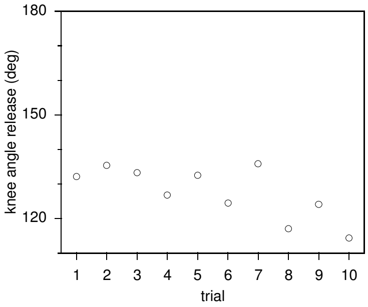

Participant 2

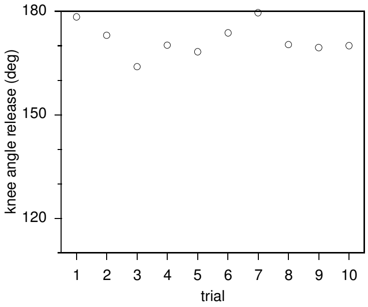

Participant 3

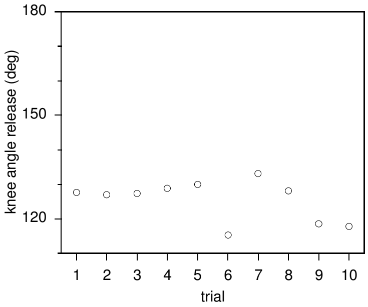

Participant 4

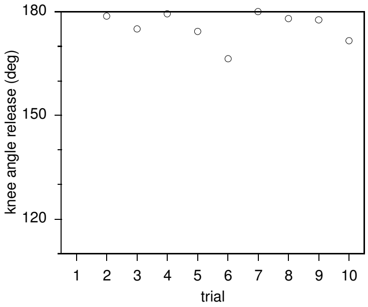

Participant 5

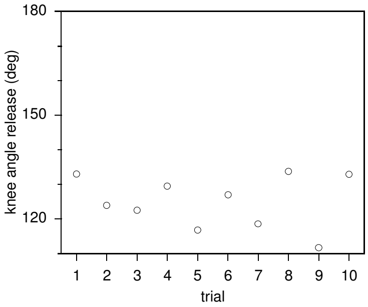

Participant 6

Participant 7

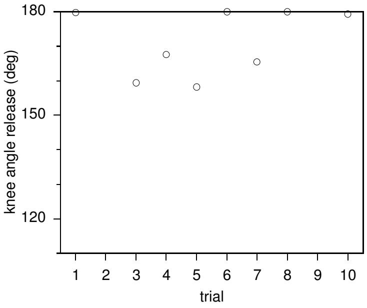

Participant 8

Participant 9

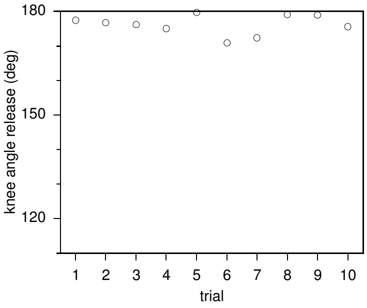

Participant 10

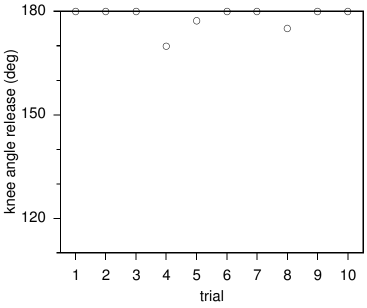

SHOULDER ANGLE X (BACK FOOT CONTACT) (AFTER)

Participant 1

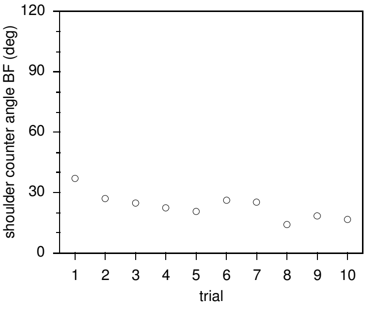

Participant 6

Participant 2

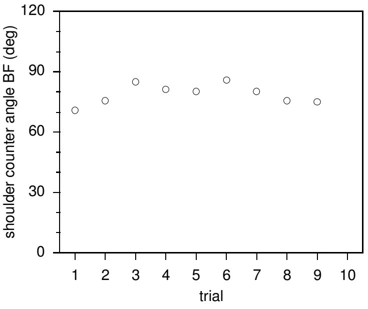

Participant 7

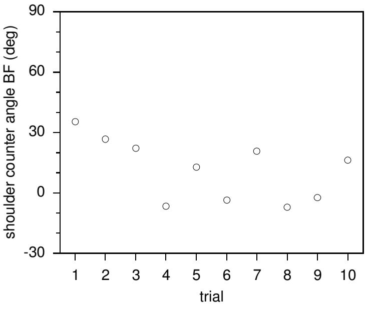

Participant 3

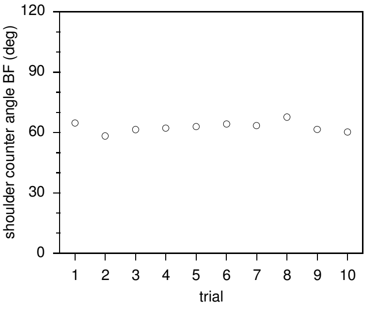

Participant 8

Participant 4

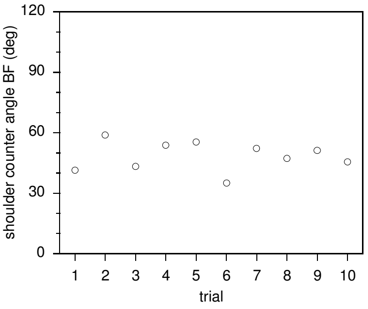

Participant 9

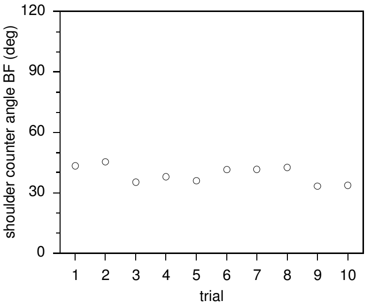

Participant 5

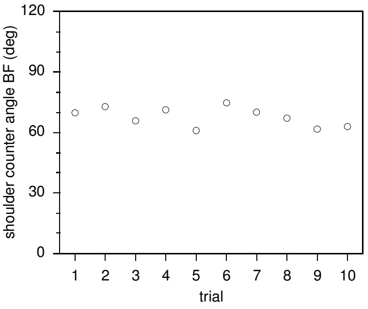

Participant 10

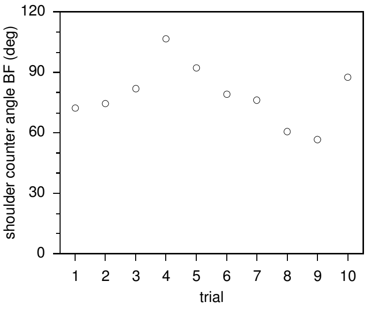

SHOULDER ANGLE X (FRONT FOOT CONTACT) (AFTER)

Participant 1

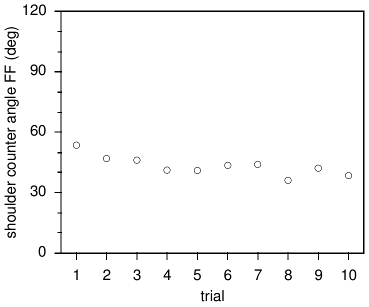

Participant 6

Participant 2

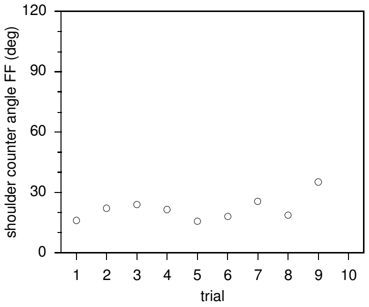

Participant 7

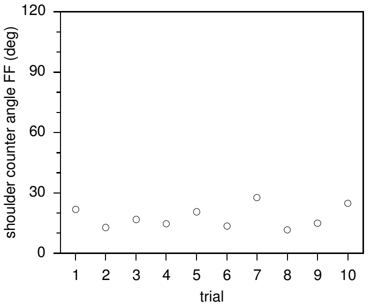

Participant 3

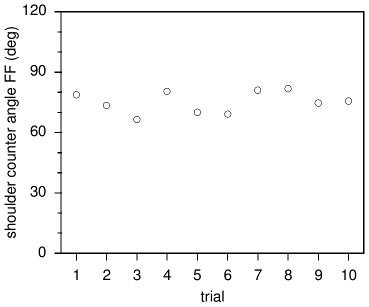

Participant 8

Participant 4

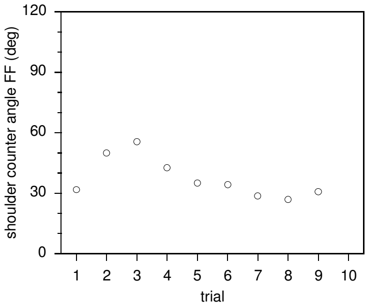

Participant 9

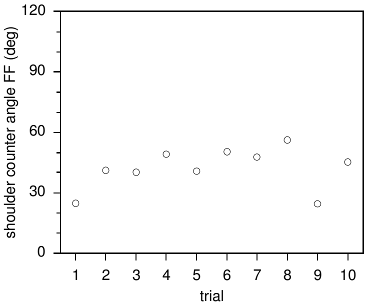

Participant 5

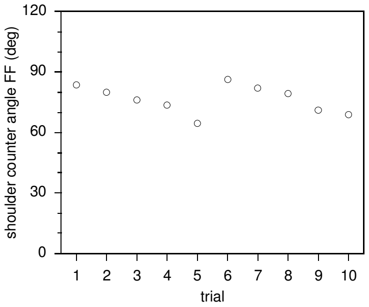

Participant 10

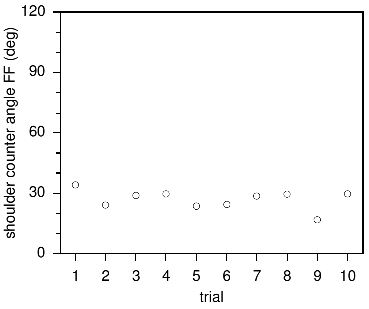

Supplement: Supplementary file 1 [file sports-05-00018-s001.zip › Supplementary Data 3 (After).pdf]
