# Supplementary material for: Effect of Ball Weight on Speed, Accuracy, and Mechanics in Cricket Fast Bowling
Source: Sports (Basel). 2017 Feb 28;5(1):18. doi: 10.3390/sports5010018 (PMC5969017; doi:10.3390/sports5010018)
Supplement: Supplementary file 1 [file sports-05-00018-s001.zip › Supplementary Data 4 (Effect of Training).pdf]

EFFECT OF TRAINING PROGRAMS (INDIVIDUAL ANALYSIS)

Ball Speed

Training group	Participant	Before (m/s)	After (m/s)	Difference (m/s)	±90% CI (m/s)	Interpretation
Intervention	1	32.6	34.7	2.1	0.9	Probably beneficial
	2	32.0	31.7	–0.3	0.3	Probably trivial
	3	30.9	32.5	1.7	0.5	Probably beneficial
	4	30.8	31.6	0.8	0.6	Possibly trivial
	5	30.6	31.2	0.6	0.2	Probably trivial
Control	6	31.6	—	—	—	—
	7	31.0	30.2	–0.8	0.4	Possibly trivial
	8	29.5	—	—	—	—
	9	28.9	29.6	0.8	0.8	Possibly trivial
	10	27.3	27.9	0.6	0.6	Probably trivial

Accuracy

Training group	Participant	Before (point)	After (point)	Difference (point)	±90% CI (point)	Interpretation
Intervention	1	1.5	2.7	1.2	0.7	Almost certainly beneficial
	2	2.0	2.7	0.7	0.6	Unclear
	3	1.7	2.7	1.0	0.6	Probably beneficial
	4	1.8	2.5	0.7	0.6	Unclear
	5	1.6	2.3	0.7	0.8	Unclear
Control	6	2.5	—	—	—	—
	7	2.2	2.2	0.0	0.5	Unclear
	8	1.4	—	—	—	—
	9	1.9	2.3	0.4	0.7	Unclear
	10	1.8	1.6	–0.2	0.4	Unclear

EFFECT OF TRAINING PROGRAMS (INDIVIDUAL ANALYSIS)

Back Foot Angle

Training group	Participant	Before (deg)	After (deg)	Difference (deg)	±90% CI (deg)	Interpretation
Intervention	1	106	109	3	5	Probably trivial
	2	40	38	–2	7	Probably trivial
	3	34	36	2	7	Probably trivial
	4	73	64	–9	4	Probably trivial
	5	59	36	–23	8	Possibly decreased
Control	6	63	—	—	—	—
	7	73	40	–33	7	Almost certainly decreased
	8	35	—	—	—	—
	9	56	76	20	5	Possibly trivial
	10	103	82	–22	8	Possibly decreased

Front Foot Angle

Training group	Participant	Before (deg)	After (deg)	Difference (deg)	±90% CI (deg)	Interpretation
Intervention	1	15	3	–12	3	Possibly trivial
	2	–14	–7	7	10	Probably trivial
	3	4	–3	–7	6	Possibly trivial
	4	–5	6	12	5	Possibly trivial
	5	–4	–1	3	6	Probably trivial
Control	6	15	—	—	—	—
	7	5	20	16	6	Probably trivial
	8	–7	—	—	—	—
	9	41	0	–41	6	Almost certainly decreased
	10	40	34	–6	10	Probably trivial

* Foot angle measured in clockwise direction relative to direction of run-up

EFFECT OF TRAINING PROGRAMS (INDIVIDUAL ANALYSIS)

Knee Angle (Back Foot Contact)

Training group	Participant	Before (deg)	After (deg)	Difference (deg)	±90% CI (deg)	Interpretation
Intervention	1	174	176	1	3	Probably trivial
	2	147	143	–4	4	Possibly trivial
	3	150	146	–4	4	Possibly trivial
	4	163	161	–3	4	Probably trivial
	5	142	141	–1	3	Probably trivial
Control	6	155	—	—	—	—
	7	154	152	–2	4	Probably trivial
	8	142	—	—	—	—
	9	149	163	14	5	Probably increased
	10	166	168	3	4	Probably trivial

Knee Angle (Front Foot Contact)

Training group	Participant	Before (deg)	After (deg)	Difference (deg)	±90% CI (deg)	Interpretation
Intervention	1	154	162	8	5	Possibly trivial
	2	159	164	5	5	Possibly trivial
	3	141	160	19	5	Almost certainly increased
	4	164	170	6	5	Possibly trivial
	5	147	148	1	6	Probably trivial
Control	6	175	—	—	—	—
	7	162	177	15	3	Probably increased
	8	156	—	—	—	—
	9	177	169	–8	3	Possibly trivial
	10	172	179	7	4	Possibly trivial

Knee Angle (Release)

Training group	Participant	Before (deg)	After (deg)	Difference (deg)	±90% CI (deg)	Interpretation
Intervention	1	132	128	–4	5	Possibly trivial
	2	177	172	–5	5	Possibly trivial
	3	131	125	–6	5	Possibly trivial
	4	179	176	–3	3	Possibly trivial
	5	128	125	–3	5	Possibly trivial
Control	6	153	—	—	—	—
	7	180	173	–7	6	Possibly trivial
	8	131	—	—	—	—
	9	176	176	1	4	Probably trivial
	10	180	178	–2	2	Possibly trivial

EFFECT OF TRAINING PROGRAMS (INDIVIDUAL ANALYSIS)

Shoulder Angle (Back Foot Contact)

Training group	Participant	Before (deg)	After (deg)	Difference (deg)	±90% CI (deg)	Interpretation
Intervention	1	37	23	–14	5	Possibly trivial
	2	78	79	1	4	Probably trivial
	3	59	63	4	2	Probably trivial
	4	49	48	0	5	Probably trivial
	5	50	68	18	4	Possibly trivial
Control	6	32	—	—	—	—
	7	51	11	–40	9	Almost certainly decreased
	8	—	—	—	—	—
	9	48	39	–9	4	Probably trivial
	10	67	79	12	10	Possibly trivial

Shoulder Angle (Front Foot Contact)

Training group	Participant	Before (deg)	After (deg)	Difference (deg)	±90% CI (deg)	Interpretation
Intervention	1	24	43	20	4	Possibly trivial
	2	17	22	5	5	Probably trivial
	3	73	75	2	4	Probably trivial
	4	17	37	20	7	Possibly trivial
	5	95	77	–18	5	Possibly trivial
Control	6	10	—	—	—	—
	7	7	18	11	4	Possibly trivial
	8	—	—	—	—	—
	9	6	42	36	7	Almost certainly increased
	10	9	27	19	4	Possibly trivial

* Foot angle measured in anti-clockwise direction relative to direction of run-up

EFFECT OF TRAINING PROGRAMS (GROUP ANALYSIS)

Intervention (n=5)

Variable	Meaningful difference	Mean difference	±90%CI	*p*-value	Cohen's *d*	Within-pair correlation	Interpretation
Ball speed (m/s)	1.5	0.99	0.87	0.07*	1.08	0.77	Possibly trivial
Accuracy (points)	0.1	0.85	0.24	<0.01*	3.38	0.21	Almost certainly beneficial
Back foot angle (deg)	20	–5.7	10.1	0.30	–0.53	0.94	Probably trivial
Front foot angle (deg)	20	0.5	9.3	0.92	0.05	0.42	Probably trivial
Back knee angle at back-foot contact (deg)	10	–2.0	2.3	0.13	–0.85	0.99	Probably trivial
Front knee angle at front-foot contact (deg)	10	7.8	6.5	0.06*	1.16	0.70	Possibly trivial
Front knee angle at ball release (deg)	10	–4.2	1.2	<0.01*	–3.41	0.99	Very likely trivial
Shoulder angle at back-foot contact (deg)	20	1.6	10.7	0.77	0.14	0.86	Probably trivial
Shoulder angle at front-foot contact (deg)	20	5.6	14.8	0.46	0.36	0.94	Possibly trivial

Control (n=3)

Variable	Meaningful difference	Mean difference	±90%CI	*p*-value	Cohen's *d*	Within-pair correlation	Interpretation
Ball speed (m/s)	1.5	0.20	1.43	0.72	0.24	0.93	Possibly trivial
Accuracy (points)	0.1	0.08	0.43	0.65	0.30	0.78	Unclear
Back foot angle (deg)	20	–11.5	19.4	0.55	–0.42	0.30	Unclear
Front foot angle (deg)	20	–10.5	48.3	0.59	–0.37	–0.16	Unclear
Back knee angle at back-foot contact (deg)	10	4.9	14.0	0.42	0.59	0.53	Possibly trivial
Front knee angle at front-foot contact (deg)	10	4.5	19.5	0.57	0.39	–0.61	Unclear
Front knee angle at ball release (deg)	10	–2.6	6.1	0.34	–0.71	–0.11	Probably trivial
Shoulder angle at back-foot contact (deg)	20	–12.1	44.0	0.51	–0.46	0.82	Unclear
Shoulder angle at front-foot contact (deg)	20	21.6	21.6	0.10	1.68	-0.62	Possibly increased

* Statistically significant at $\alpha = 0.10$
