# Supplementary material for: Effect of Ball Weight on Speed, Accuracy, and Mechanics in Cricket Fast Bowling
Source: Sports (Basel). 2017 Feb 28;5(1):18. doi: 10.3390/sports5010018 (PMC5969017; doi:10.3390/sports5010018)
Supplement: Supplementary file 1 [file sports-05-00018-s001.zip › Supplementary Data 1 (Ball Weight).pdf]

BALL SPEED

Participant 1

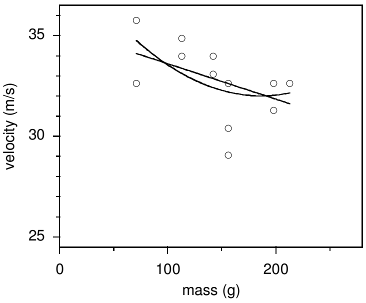

Participant 2

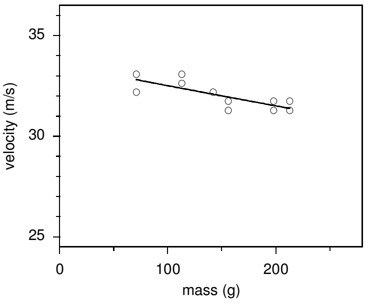

Participant 3

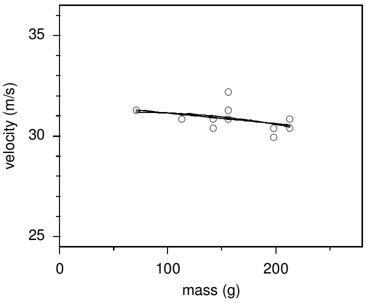

Participant 4

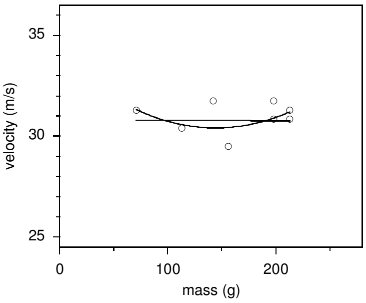

Participant 5

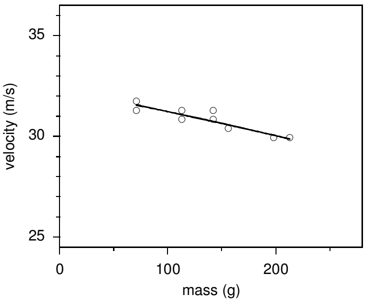

Participant 6

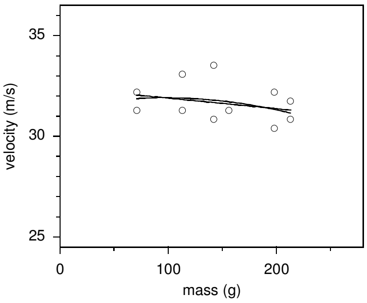

Participant 7

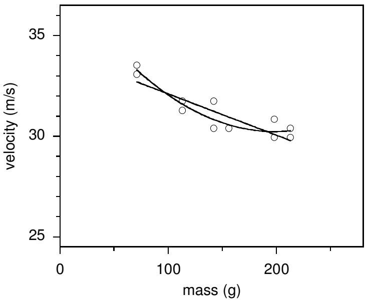

Participant 8

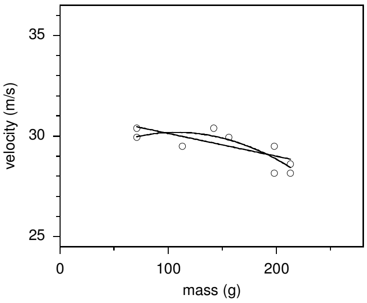

Participant 9

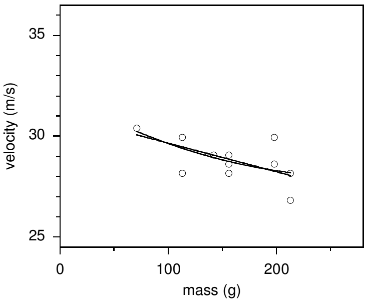

Participant 10

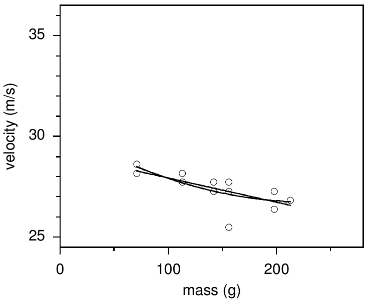

ACCURACY

Participant 1

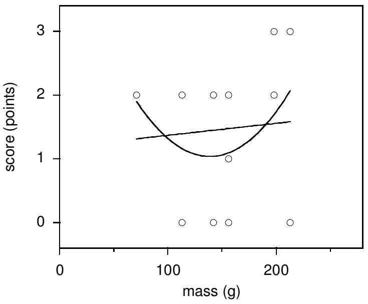

Participant 2

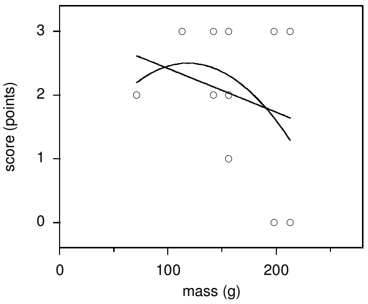

Participant 3

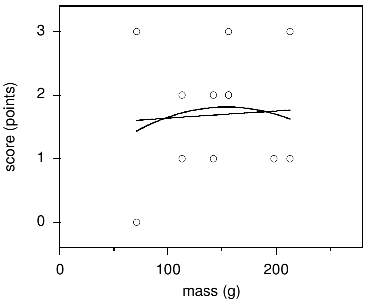

Participant 4

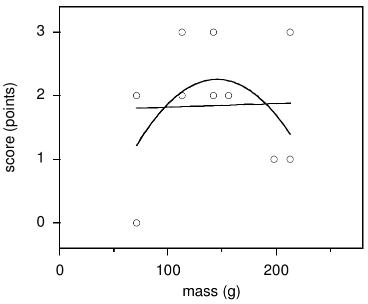

Participant 5

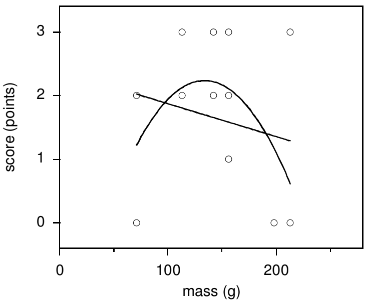

Participant 6

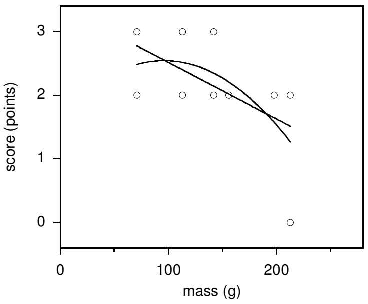

Participant 7

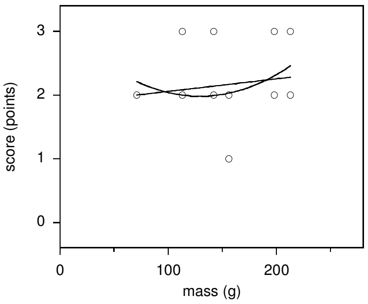

Participant 8

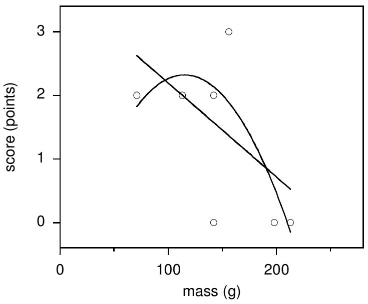

Participant 9

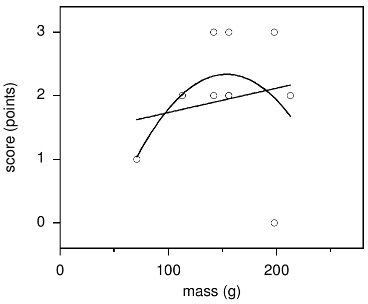

Participant 10

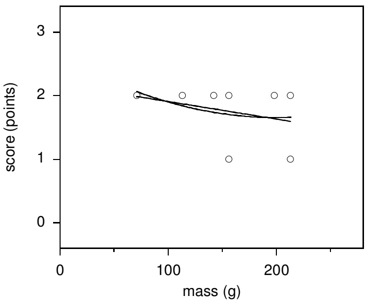

BACK FOOT ANGLE *

Participant 1

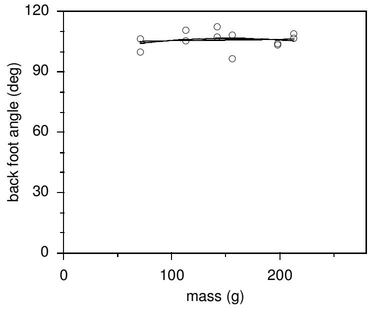

Participant 2

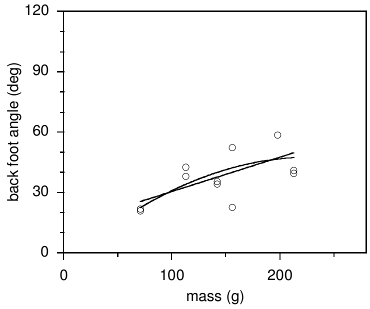

Participant 3

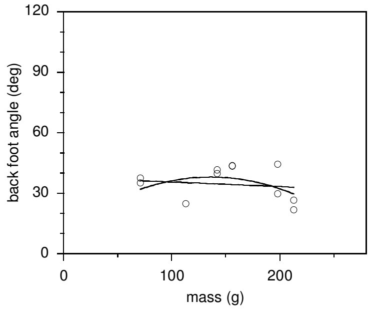

Participant 4

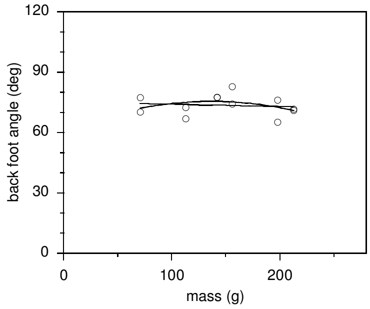

Participant 5

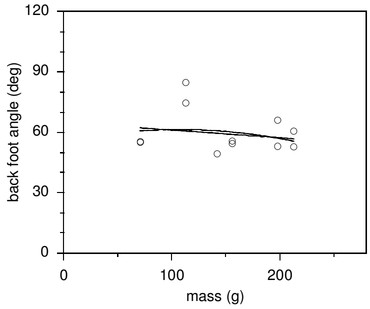

Participant 6

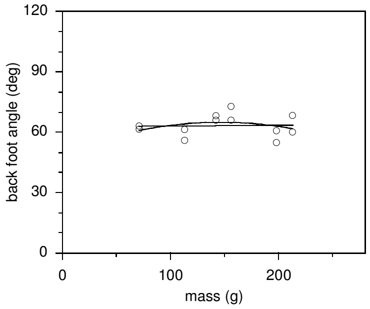

Participant 7

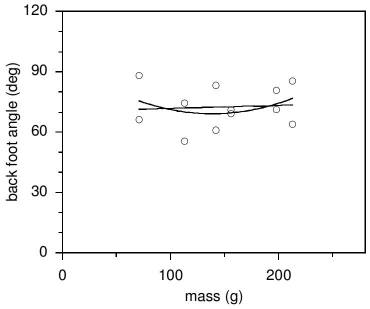

Participant 8

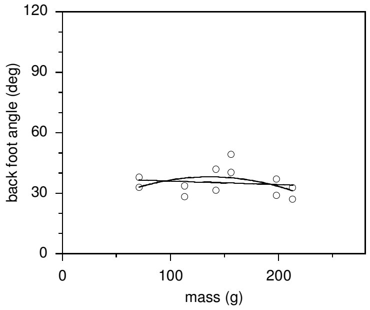

Participant 9

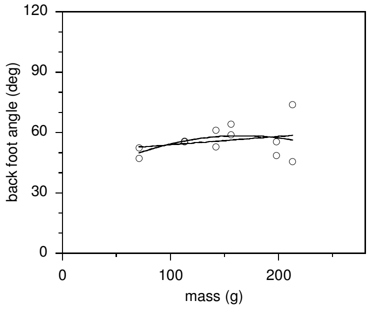

Participant 10

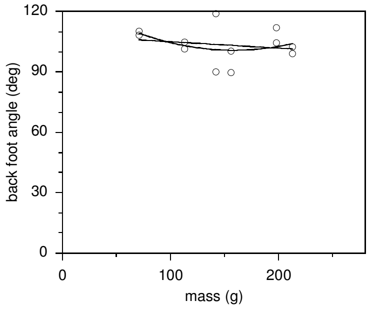

* Foot angle measured in clockwise direction

FRONT FOOT ANGLE *

Participant 1

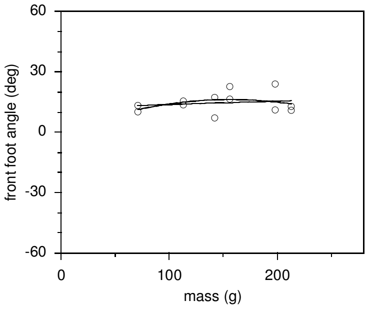

Participant 2

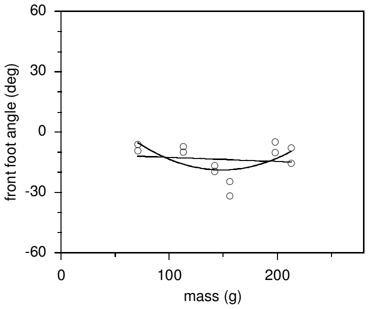

Participant 3

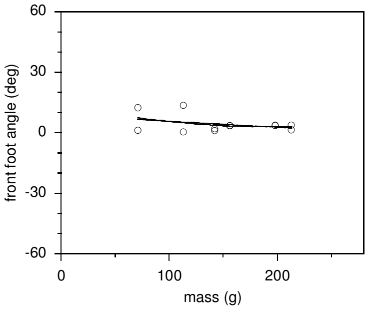

Participant 4

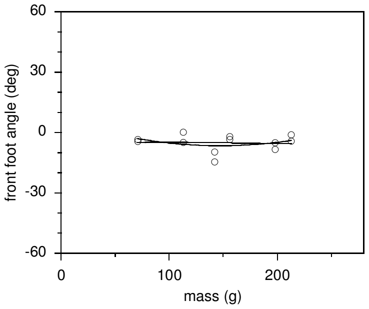

Participant 5

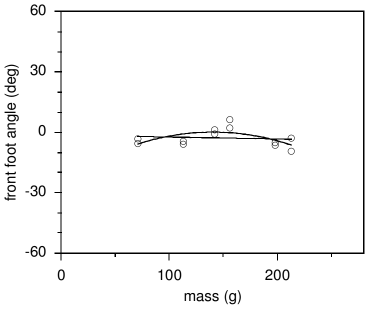

Participant 6

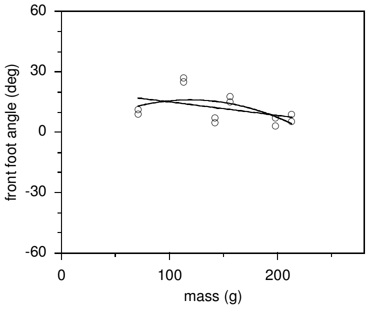

Participant 7

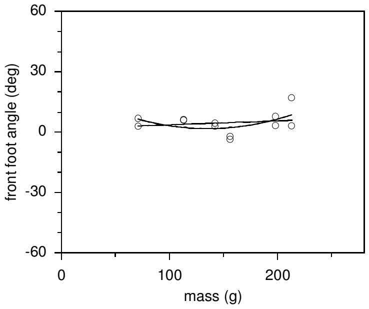

Participant 8

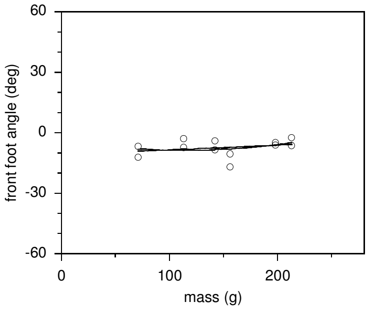

Participant 9

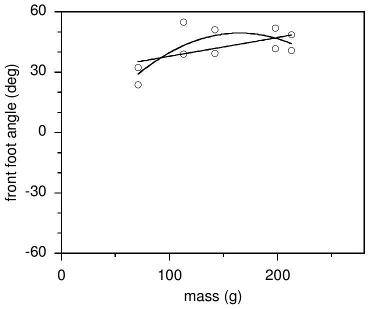

Participant 10

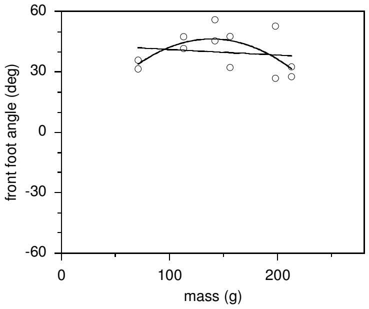

* Foot angle measured in clockwise direction

KNEE ANGLE (BACK FOOT CONTACT)

Participant 1

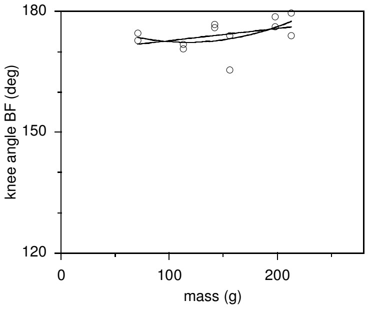

Participant 2

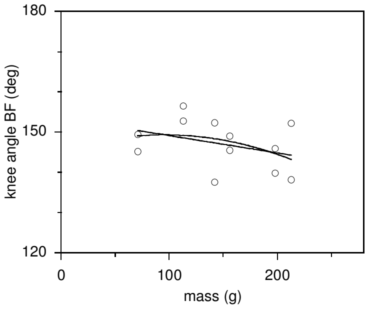

Participant 3

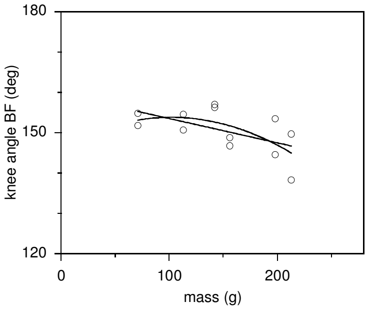

Participant 4

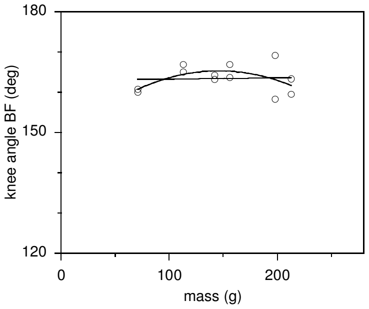

Participant 5

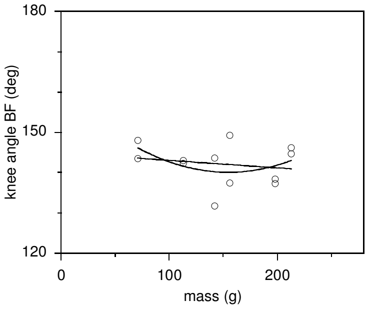

Participant 6

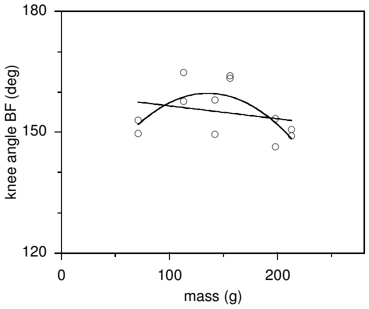

Participant 7

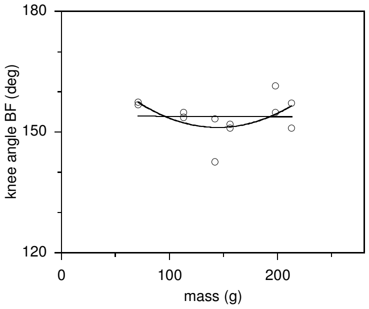

Participant 8

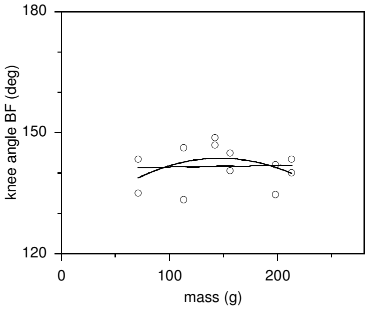

Participant 9

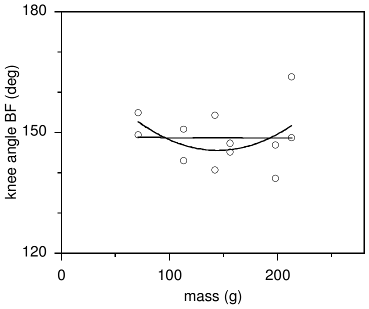

Participant 10

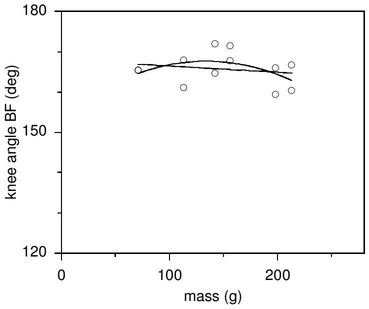

KNEE ANGLE (FRONT FOOT CONTACT)

Participant 1

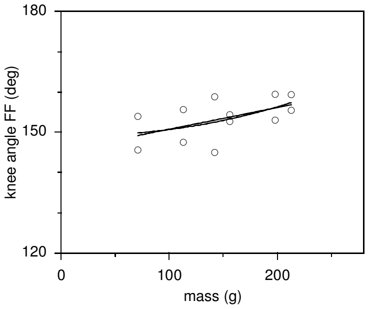

Participant 2

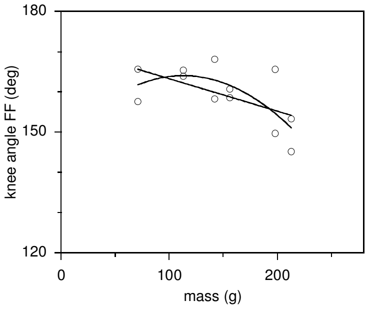

Participant 3

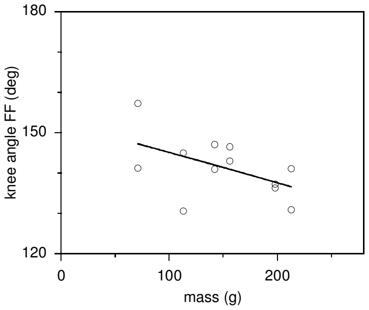

Participant 4

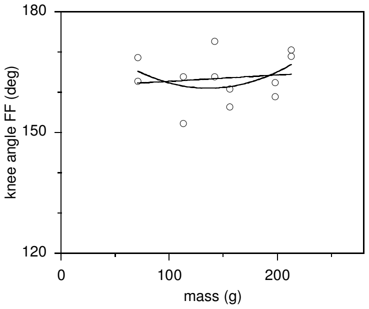

Participant 5

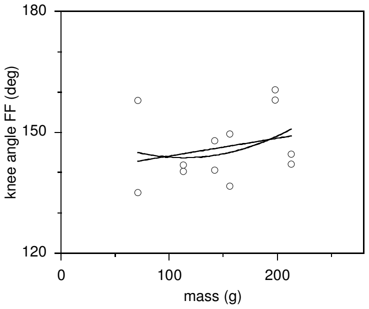

Participant 6

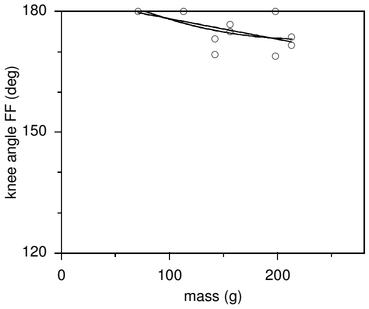

Participant 7

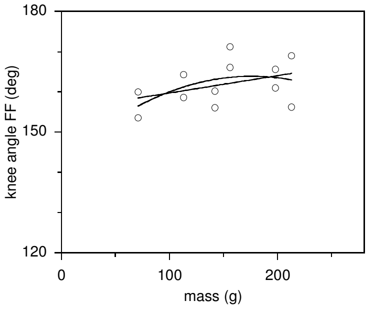

Participant 8

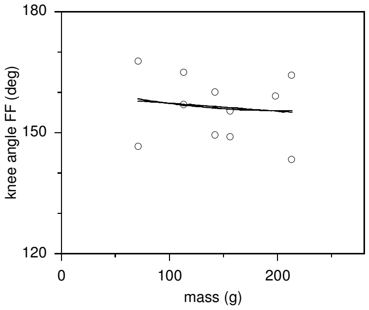

Participant 9

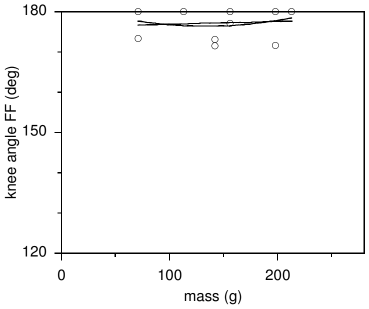

Participant 10

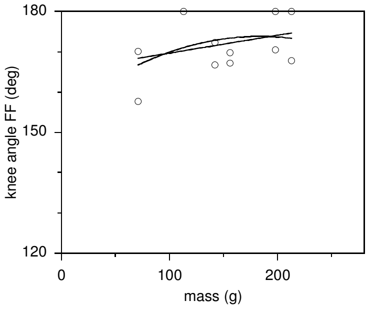

KNEE ANGLE (RELEASE)

Participant 1

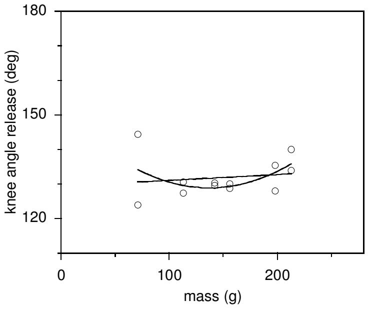

Participant 2

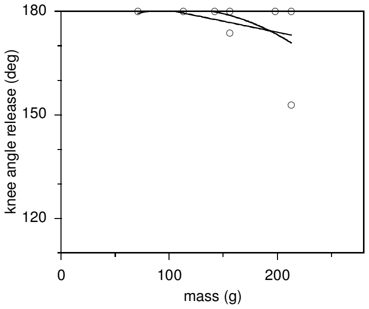

Participant 3

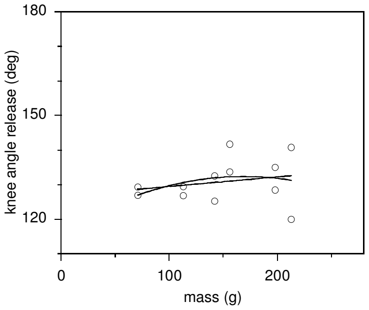

Participant 4

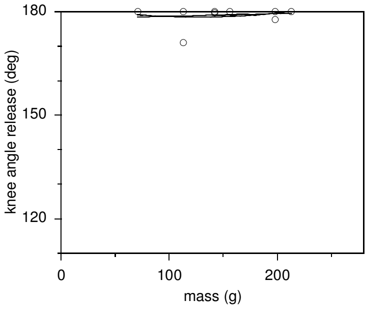

Participant 5

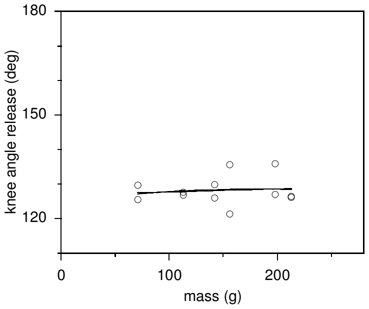

Participant 6

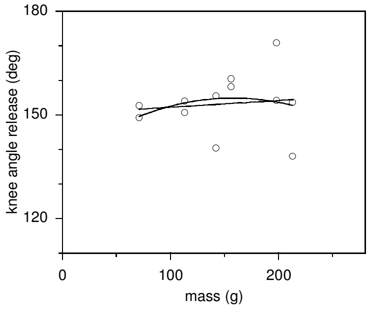

Participant 7

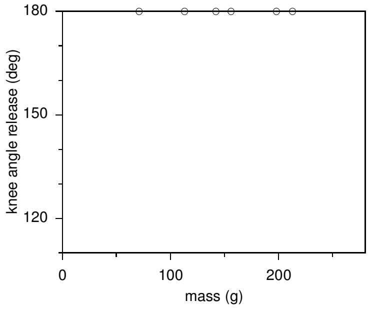

Participant 8

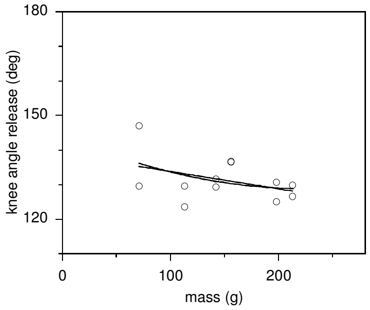

Participant 9

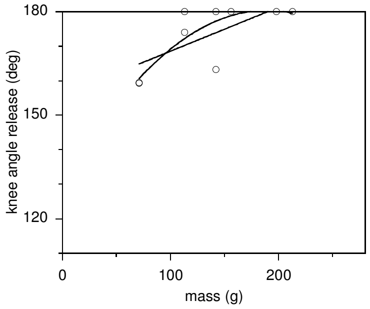

Participant 10

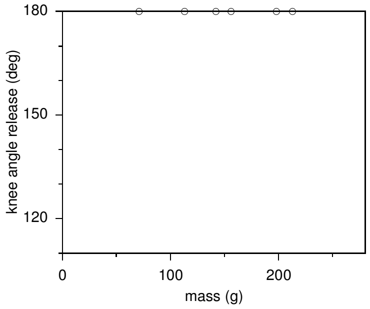

SHOULDER ANGLE X (BACK FOOT CONTACT)

Participant 1

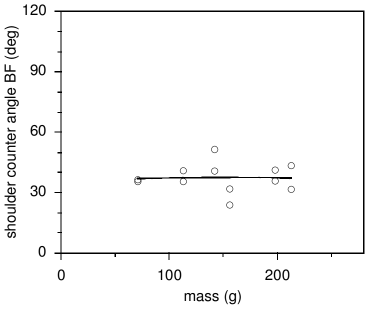

Participant 6

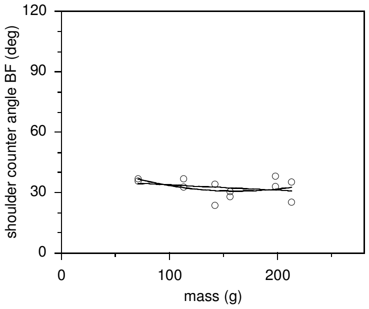

Participant 2

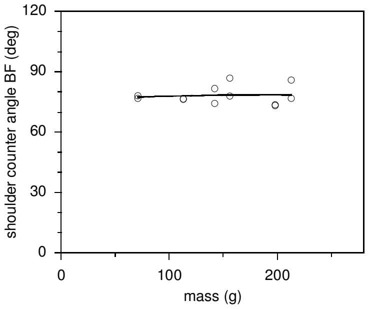

Participant 7

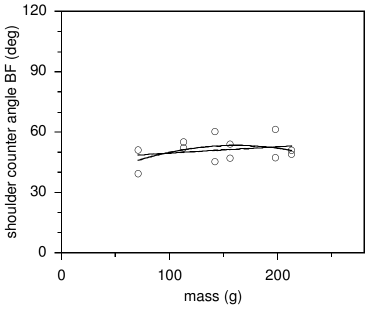

Participant 3

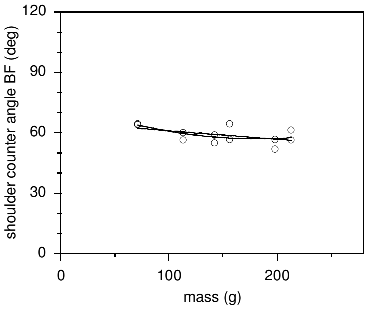

Participant 8

Participant 4

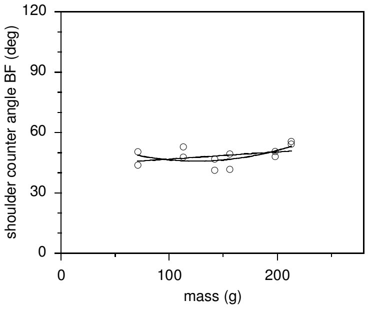

Participant 9

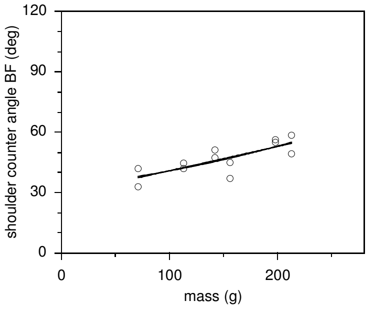

Participant 5

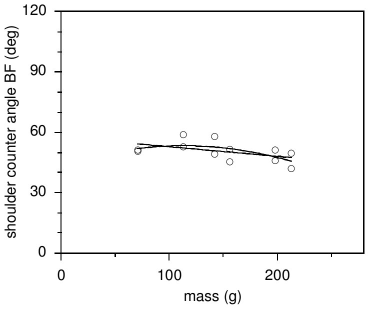

Participant 10

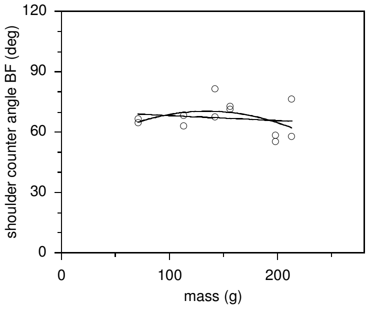

SHOULDER ANGLE X (FRONT FOOT CONTACT)

Participant 1

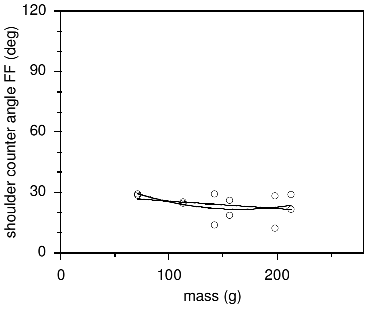

Participant 2

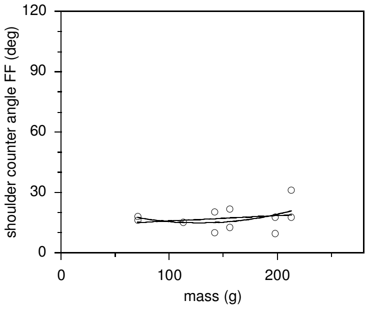

Participant 3

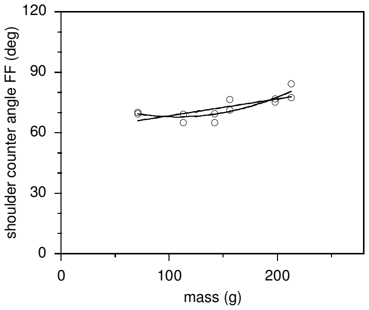

Participant 4

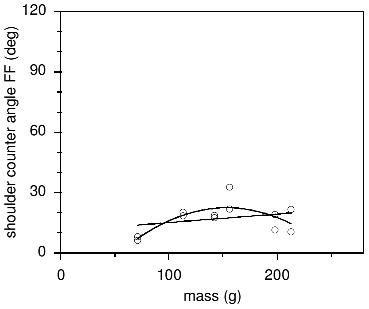

Participant 5

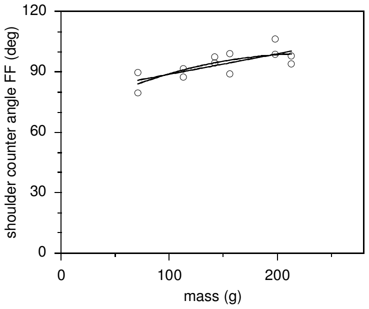

Participant 6

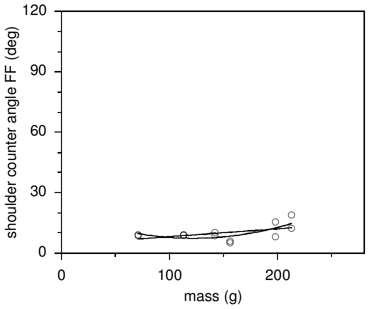

Participant 7

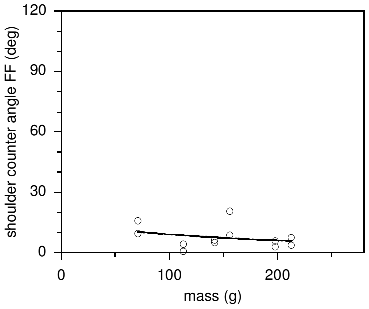

Participant 8

Participant 9

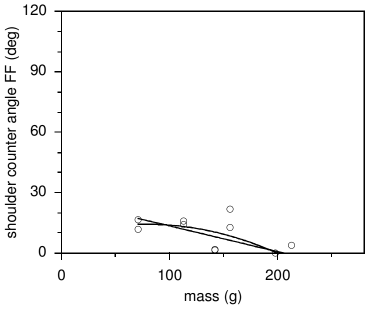

Participant 10

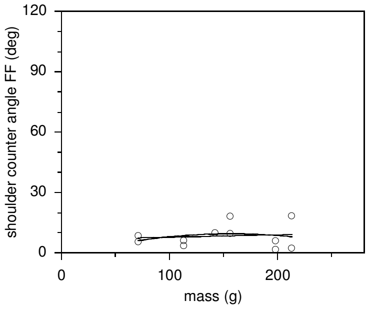

RELATIVE PROBABILITY OF LINEAR FIT OR U-SHAPE FIT

Ball Speed

Training group	Participant	Probability Linear Fit (%)	Probability U-Shape Fit (%)	Evidence Ratio
Intervention	1	83	17	4.9
	2	90	10	8.7
	3	88	12	7.4
	4	68	32	2.1
	5	89	11	8.1
Control	6	88	12	7.6
	7	6	94	0.1
	8	30	70	0.4
	9	88	12	7.4
	10	85	15	5.7

Accuracy

Training group	Participant	Probability Linear Fit (%)	Probability U-Shape Fit (%)	Evidence Ratio
Intervention	1	77	23	3.3
	2	84	16	5.1
	3	89	11	7.3
	4	64	36	1.8
	5	64	36	1.8
Control	6	80	20	1.4
	7	86	14	6.3
	8	47	53	0.9
	9	58	42	1.4
	10	89	11	7.8

RELATIVE PROBABILITY OF LINEAR FIT OR U-SHAPE FIT

Back Foot Angle

Training group	Participant	Probability Linear Fit (%)	Probability U-Shape Fit (%)	Evidence Ratio
Intervention	1	90	10	8.7
	2	89	11	7.7
	3	80	20	4.1
	4	83	17	4.8
	5	91	9	9.8
Control	6	85	15	5.7
	7	86	14	5.9
	8	78	22	3.5
	9	87	13	6.5
	10	86	14	6.1

Front Foot Angle

Training group	Participant	Probability Linear Fit (%)	Probability U-Shape Fit (%)	Evidence Ratio
Intervention	1	85	15	5.7
	2	39	61	0.6
	3	90	10	9.1
	4	84	16	5.3
	5	74	26	2.9
Control	6	77	23	3.3
	7	66	34	1.9
	8	89	11	8.1
	9	88	12	7.4
	10	39	61	0.6

RELATIVE PROBABILITY OF LINEAR FIT OR U-SHAPE FIT

Knee Angle (Back Foot Contact)

Training group	Participant	Probability Linear Fit (%)	Probability U-Shape Fit (%)	Evidence Ratio
Intervention	1	82	18	4.6
	2	90	10	8.8
	3	82	18	4.7
	4	56	44	1.3
	5	80	20	4.0
Control	6	24	76	0.3
	7	56	44	1.3
	8	81	20	4.3
	9	76	24	3.2
	10	75	25	2.9

Knee Angle (Front Foot Contact)

Training group	Participant	Probability Linear Fit (%)	Probability U-Shape Fit (%)	Evidence Ratio
Intervention	1	91	9	9.7
	2	66	34	1.9
	3	91	9	10.6
	4	81	19	4.3
	5	89	11	8.4
Control	6	89	11	8.2
	7	86	14	6.1
	8	91	9	10.4
	9	89	11	8.3
	10	90	10	8.5

Knee Angle (Release)

Training group	Participant	Probability Linear Fit (%)	Probability U-Shape Fit (%)	Evidence Ratio
Intervention	1	69	31	2.2
	2	86	14	6.2
	3	89	11	8.2
	4	89	11	8.4
	5	91	9	10.3
Control	6	90	10	8.6
	7	100	0	>100
	8	100	0	>100
	9	59	41	1.4
	10	100	0	>100

RELATIVE PROBABILITY OF LINEAR FIT OR U-SHAPE FIT

Shoulder Angle (Back Foot Contact)

Training group	Participant	Probability Linear Fit (%)	Probability U-Shape Fit (%)	Evidence Ratio
Intervention	1	91	9	10.4
	2	91	9	10.3
	3	82	18	4.6
	4	65	35	1.8
	5	77	23	3.4
Control	6	82	18	4.6
	7	84	16	5.1
	8	–	–	–
	9	91	9	10.4
	10	80	20	4.0

Shoulder Angle (Front Foot Contact)

Training group	Participant	Probability Linear Fit (%)	Probability U-Shape Fit (%)	Evidence Ratio
Intervention	1	84	16	5.1
	2	64	36	1.8
	3	25	75	0.3
	4	12	88	0.1
	5	86	14	6.2
Control	6	47	53	0.9
	7	91	9	10.3
	8	–	–	–
	9	85	15	5.6
	10	89	11	8.4

GRADIENT OF LINEAR FIT

Ball Speed

Training group	Participant	Gradient (m/s per kg)	±90% CI
Intervention	1	–18	18
	2	–10	4
	3	–5	6
	4	0	10
	5	–12	3
Control	6	–5	10
	7	–21	6
	8	–11	6
	9	–14	9
	10	–12	7

Accuracy

Training group	Participant	Gradient (points per kg)	±90% CI
Intervention	1	2	13
	2	–7	12
	3	1	11
	4	1	10
	5	–5	14
Control	6	–9	7
	7	2	8
	8	–15	12
	9	4	13
	10	–3	5

GRADIENT OF LINEAR FIT

Back Foot Angle

Training group	Participant	Gradient (deg per kg)	±90% CI
Intervention	1	7	51
	2	171	112
	3	–23	95
	4	–11	57
	5	–38	120
Control	6	3	59
	7	15	116
	8	–18	73
	9	41	87
	10	–32	94

Front Foot Angle

Training group	Participant	Gradient (deg per kg)	±90% CI
Intervention	1	17	56
	2	–21	93
	3	–30	46
	4	–4	46
	5	–13	32
Control	6	–67	81
	7	20	58
	8	23	46
	9	88	97
	10	–28	112

GRADIENT OF LINEAR FIT

Knee Angle (Back Foot Contact)

Training group	Participant	Gradient (deg per kg)	±90% CI
Intervention	1	30	40
	2	–43	65
	3	–60	51
	4	3	38
	5	–19	56
Control	6	–32	71
	7	–1	53
	8	4	48
	9	–2	78
	10	–15	44

Knee Angle (Front Foot Contact)

Training group	Participant	Gradient (deg per kg)	±90% CI
Intervention	1	54	51
	2	–81	66
	3	–75	40
	4	16	68
	5	44	95
Control	6	–51	40
	7	43	57
	8	–20	88
	9	7	42
	10	44	76

Knee Angle (Release)

Training group	Participant	Gradient (deg per kg)	±90% CI
Intervention	1	17	70
	2	–64	82
	3	28	70
	4	8	29
	5	9	47
Control	6	19	97
	7	0	0
	8	–50	66
	9	127	68
	10	0	0

GRADIENT OF LINEAR FIT

Shoulder Angle (Back Foot Contact)

Training group	Participant	Gradient (deg per kg)	±90% CI
Intervention	1	1	79
	2	8	50
	3	–42	39
	4	36	49
	5	–48	48
Control	6	–26	52
	7	31	68
	8	–	–
	9	121	55
	10	–25	88

Shoulder Angle (Front Foot Contact)

Training group	Participant	Gradient (deg per kg)	±90% CI
Intervention	1	–37	66
	2	27	67
	3	83	44
	4	43	79
	5	102	53
Control	6	39	38
	7	–31	62
	8	–	–
	9	–128	79
	10	12	62
